# Neural correlates of the attention training technique as used in metacognitive therapy – A randomized sham-controlled fMRI study in healthy volunteers

**DOI:** 10.3389/fpsyg.2023.1084022

**Published:** 2023-03-13

**Authors:** Niklas Jahn, Christopher Sinke, Özlem Kayali, Svenja Krug, Erik Leichter, Stephanie Peschel, Torben Müller, Alev Burak, Tillmann H. C. Krüger, Kai G. Kahl, Ivo Heitland

**Affiliations:** ^1^Department of Psychiatry, Social Psychiatry and Psychotherapy, Hannover Medical School, Hanover, Germany; ^2^Department of Psychiatry, Social Psychiatry and Psychotherapy, Division of Clinical Psychology and Sexual Medicine, Hannover Medical School, Hanover, Germany; ^3^Center for Systems Neuroscience, Hanover, Germany

**Keywords:** attention training technique, metacognitive therapy, attentional control, healthy participants, fMRI, anterior cingulate cortex

## Abstract

**Introduction:**

The Attention Training Technique (ATT) developed as part of metacognitive therapy is a psychotherapeutic treatment method used to enhance top-down attentional flexibility and control. This study investigated potential neurocognitive changes due to ATT and its underlying neural mechanisms using pre-to-post functional magnetic resonance imaging (fMRI).

**Materials and methods:**

Fifty-four healthy participants were subjected to a randomized, sham-controlled attention training and evaluated using a neurocognitive test battery that partly took place in an fMRI environment. Participants received two doses ATT or sham ATT daily for 1 week. On day eight, all subjects completed the neurocognitive test battery again.

**Results:**

After the training, the ATT group showed a significant improvement in reaction times regarding attentional disengagement compared to the sham ATT group. fMRI data showed decreased levels of activation in the anterior cingulate cortex (ACC) when comparing the ATT group to the sham ATT group during attentional disengagement post intervention. No ATT > sham ATT effects were found regarding selective auditory attention, working memory performance and inhibitory control.

**Discussion:**

These findings putatively indicate that ATT facilitates faster attention allocation and increased attentional flexibility in healthy subjects. The fMRI results suggest this ATT-dependent improvement is accompanied by reduced ACC activity, indicating a more flexible attentional state.

## Introduction

The Attention Training Technique (ATT) is a psychotherapeutic treatment method originally developed as part of metacognitive therapy (MCT; Wells, 1990). The theoretical foundation of MCT is based on the Self-Regulatory Executive Function Model (S-REF; Wells and Matthews, 1996), which depicts the elements involved in the regulation and control of our cognitive mechanisms in a three-level system: low-level automatic cognitive processing, conscious, deliberate processing, and the metacognitive system. While metacognition fundamentally encompasses any knowledge, belief or cognitive process involved in cognitive monitoring or cognitive control (Flavell, 1979; Kitchener, 1983), as per S-REF theory, metacognition can be further subdivided into several components represented in the metacognitive control system: metacognitive knowledge (declarative, verbally expressible knowledge and procedural knowledge, which represents plans directing thought processes), cybernetic code (the current status of cognition as an internal code used to alter and regulate neural networks), and structures that support goal directed processing including a comparator mechanism constantly comparing the cognitive style of processing with an internal model (Wells, 2019). These different and interdependent metacognitive constituents regulate, control, and monitor our conscious thinking and, thus, are responsible for one’s respective cognitive style. According to the S-REF model, a maladaptive metacognitive system with biased metacognitive beliefs and knowledge, which persists in patients with psychological disorders, can induce a certain unhelpful thinking pattern called the Cognitive Attentional Syndrome (CAS). These erroneous metacognitive beliefs can be positive as well as negative in nature, but both contribute to the CAS. Positive metacognitive beliefs support unhelpful coping strategies by focussing on their ostensible benefits (e.g., “Worrying will help me in order to know what to do.”), whereas negative metacognitive beliefs concern biased knowledge about the uncontrollability and harmfulness of thoughts (e.g., “My thoughts will make me do something.”; Wells, 2009). The CAS consists of perseverative thought processes including worry and rumination as well as threat monitoring. Furthermore, the CAS includes detrimental coping strategies like suppression of thoughts, avoidance behavior or substance abuse (Wells, 2009). It is characterized by inflexible, self-focused attention and, thus, causes aversive emotions to be prolonged and intensified, leading to a constant state of negative self-processing and emotional distress. This dysfunctional pattern is assumed to cause and maintain psychological disorders.

Repetitive negative thinking as part of the CAS is associated with adverse emotional outcomes (Nolen-Hoeksema, 1991; Ehring and Watkins, 2008). Capobianco et al. (2018) found initial evidence for a negative impact on stress recovery due to repetitive negative thinking, whereas Trick et al. (2019) found that rumination and poor problem-solving skills predict depression in patients with acute coronary syndrome. These results demonstrate the need for a treatment of the underlying problem.

MCT is designed to tackle this dysfunctional thinking style. It aims to help the patient exit this loop of repetitive negative thinking by means of various methods, one of which is the Attention Training Technique, a cornerstone of the MCT.

ATT is used in order to reduce or prevent CAS activation by enhancing metacognitive control (Knowles and Wells, 2018). It aims to improve the disengagement from perseverative thinking processes and to disrupt self-focused attention (Fergus and Wheless, 2018) by modifying attentional flexibility (Knowles et al., 2016) and increasing metacognitive awareness (Nassif and Wells, 2014). ATT was designed in order to impact and reinforce one’s metacognitive system and improve flexible executive control *via* attentional redirection (Wells, 2009). Low attentional control seems to be associated with a stronger link between CAS activation and psychopathological symptoms (Fergus et al., 2012). Furthermore, Murray et al. (2018) showed that, children delayed gratification significantly longer following ATT in comparison to progressive muscle relaxation or no intervention, indicating an improvement in executive control by means of ATT.

ATT has been used as a treatment of various psychological disorders. Callinan et al. (2014) and Nassif and Wells (2014) investigated the impact of ATT on traumatic stress symptoms. Subjects in both studies not only reported significantly less intrusions, but also experienced improved attentional flexibility according to self-reports. In their study, Myhr et al. (2019) found a reduction of perceived stress and meta-worry among stressed students post ATT intervention in comparison to a control group, whereas Stewart et al. (2021) did not see any significant results when examining worry and worry-related processes of patients with probable general anxiety disorder after an ATT intervention in comparison to a control group. Furthermore, ATT has been observed to reduce hypervigilance to sensory pain words (Sharpe et al., 2010). Fergus et al. (2014), Haukaas et al. (2018), and McEvoy et al. (2017) all investigated ATT in comparison to a mindfulness based intervention. Fergus et al. (2014) observed a reduction in anxiety in both groups, with the ATT group showing less self-focused attention. Although participants in the study conducted by McEvoy et al. (2017) also showed changes in anxiety, a shift in attentional focus was not replicated after a single session of ATT. Significant symptom relief regarding depression and anxiety as well as heightened attention flexibility was also seen across both groups in Haukaas et al. (2018) concluding an improvement in attentional flexibility as essential for both mechanisms.

Despite this growing body of research regarding clinical effects of ATT, data about the neurophysiological mechanisms of this treatment method is scarce as of yet. In a study conducted by Siegle et al. (2007), six depressed participants received “cognitive control training” (CCT), an intervention involving ATT and a variation of the Paced Auditory Serial Addition Task (PASAT; Gronwall, 1977). Pre-to-post fMRI analysis showed elevated dorsolateral prefrontal cortex (DLPFC) activity during a cognitive task and decreased amygdala activity during an emotional task. Kowalski et al. (2020) investigated modulation of neural connectivity through ATT (vs. a control condition) in high-and low-CAS individuals using fMRI. They observed differences in cerebral activity in multiple attention-oriented brain regions during ATT compared to a control condition, including the dorsal attention network (DAN), the fronto-parietal network (FPN) and the default mode network (DMN). They also saw reduced connectivity in the FPN during rumination induction sessions after an ATT intervention in comparison to a control condition. The extent of these results differed between high-and low-CAS individuals. Recently, Rosenbaum et al. (2018) used functional near-infrared spectroscopy (fNIRS) to analyze changes in blood oxygenation during ATT. The right inferior frontal gyrus (IFG), the right dorsolateral prefrontal cortex (DLPFC) and the superior parietal lobule (SPL) showed elevated levels of blood oxygenation during the ATT condition in contrast to a passive white noise control condition. In addition, using electroencephalography (EEG) Knowles and Wells (2018) observed enhanced resting alpha and beta-band activity in frontoparietal regions after a single dose of ATT. These findings suggest that the neuronal underpinnings of ATT can be found in the cognitive control network (CCN; Cole and Schneider, 2007) and the dorsal attention network (DAN; Vossel et al., 2014). The cognitive control network, a neural network with high functional connectivity (Cole and Schneider, 2007) frequently responding during attention demanding tasks, consists of multiple attention-related brain regions including the anterior cingulate cortex (ACC), the DLPFC and the posterior parietal cortex (PPC). The ACC is generally associated with attentional control (Botvinick et al., 2001), including conflict monitoring (Van Veen and Carter, 2002; Botvinick et al., 2004) as well as top-down attentional control regarding the processing of sensory information (Crottaz-Herbette and Menon, 2006).

Although research regarding the neuronal underpinnings of the ATT itself is limited, there are a growing number of studies investigating ATT related mechanisms. For instance, the three attentional components of the ATT (selective attention, attentional switching and divided attention) were all subject to several studies regarding visual as well as auditory attention. Salmi et al. (2007) discovered increased bilateral activation in the superior parietal lobule, the middle frontal gyrus (MFG), the temporoparietal junction (TPJ) and the superior frontal gyrus (SFG) during an attention-orienting task for auditory as well as visual attention. According to the findings of Hanlon et al. (2017), the ventrolateral prefrontal cortex (VLPFC) could be partly responsible for fast attention deployment. Also, in contrast to earlier studies, they did not find any evidence for a difference in activation in the dorsal and ventral frontoparietal networks during orienting and reorienting trials. Furthermore, the posterior parietal cortex is involved in switching attention between auditory stimuli (Shomstein and Yantis, 2006; Lee et al., 2014). In addition, Moisala et al. (2015) employed a congruence judgment task in order to examine the neuronal differences between selective and divided attention. Their findings suggest that divided attention does not demand additional brain areas, but showed enhanced activity in medial and lateral frontal regions in comparison to selective attention.

These studies beg the question which attention-related cerebral regions are ultimately affected by ATT and are responsible for its promising behavioral and therapeutic results. Therefore, the main objective of this study was to follow up on this line of research by (1) investigating the neurophysiological changes caused by the ATT and (2) to further examine the attentional domains affected by ATT using pre-to-post fMRI.

To that end, we implemented two fMRI tasks focused on attentional processes as well as cross-modality transfer effects: the emotional dot probe task (MacLeod et al., 1986) and the Stroop task (Stroop, 1935). Several studies using spatial attention or dot probe tasks showed an increased activity in overlapping dorsal frontal and parietal regions (Armony and Dolan, 2002, fear conditioning with neutral vs. fearful faces; Pourtois et al., 2006, neutral vs. fearful face dot probe; Slagter et al., 2007, spatial attention task with location and color cues). Furthermore, the underlying mechanisms of distraction, interference and attentional control have been investigated multiple times using Stroop-like tasks, identifying three main brain regions: the ACC, the DLPFC and the PPC (Bush et al., 1998; Banich et al., 2000; Milham et al., 2001).

Moreover, this study is a follow-up study of Barth et al. (2019), which concluded an increase in attentional flexibility in a healthy student sample. Given the current replication crisis in psychology (Maxwell et al., 2015), we additionally aimed to replicate the previous findings by Barth et al. (2019) using a non-student sample as a more representative group. Therefore, we also implemented the dichotic listening task as a measurement for selective attention in the auditory domain and as a near-transfer task and the 2-back task as a measurement for working memory performance as in the original study. This cohort will be used as the control group for future studies with different patient cohorts, which are currently conducted in our lab. The intervention interval was set to 1 week since we aimed to investigate short-term effects due to ATT as a proof-of-concept and for investigating short-term neurocognitive changes.

Based on previous findings, activity changes in multiple attention-related regions were expected. (1) With regard to aforementioned studies on the neuronal underpinnings of ATT (Knowles and Wells, 2018; Rosenbaum et al., 2018; Kowalski et al., 2020) and general knowledge about the visual (Corbetta et al., 2008) and auditory (Lee et al., 2014) attentional networks, we expected to see decreased pre-to-post activation in the FPN and/or the CCN network in the ATT group in comparison to a sham ATT group. (2) In theory, ATT should improve the ability to disengage from distracting stimuli. Thus, it was hypothesized that the ATT group would show a greater improvement in all disengagement demanding trials in the emotional dot probe task and in accordance with this, altered attentional network activity. Specifically, decreased ACC and superior/inferior parietal lobule activity was expected. The left SPL and the ACC as crucial parts in the aforementioned networks were therefore chosen for a region-of-interest analysis. (3) Although the Stroop task also can be defined as an attention bias task, we did not expect any significant results, since the results of Barth et al. (2019) already failed to show a significant ATT-dependent effect in a healthy student sample. (4) Furthermore, despite altered experimental conditions for two tasks due to the use of fMRI as a measurement method and a wider sample range regarding age and educational status, a replication of the performance data of Barth et al. (2019) was expected.

## Materials and methods

### Subjects

Fifty-four healthy participants were recruited *via* an advertisement on the intranet of the Hannover Medical School. Inclusion criteria were: verbally self-reported right-handedness, age between 18 and 50 years and fluency in German. Exclusion criteria were: diagnosed psychiatric conditions verified *via* a SCID conducted by a trained experimenter, neurological conditions, psychotropic medication, opioids or other drug abuse/intake, pregnancy as well as other grave health or social problems that would interfere with the study participation. FMRI-specific exclusion criteria were: pregnancy, stents, claustrophobia, tinnitus or metal implants. No subject reported any current or past psychiatric ICD-10 diagnosis as confirmed by means of a structured clinical interview for DSM-IV (SCID). All study procedures were in accordance with the Declaration of Helsinki (World Medical Association, 1964) and were approved by the local ethics committee of Hannover Medical School. All subjects gave their written informed consent prior to participation and received financial compensation.

The study sample is part of a larger recruitment process including the aforementioned studies by Barth et al. (2019) and Heitland et al. (2020), resulting in an overlap between this sample and the Heitland et al. (2020) sample.

**Figure 1:**
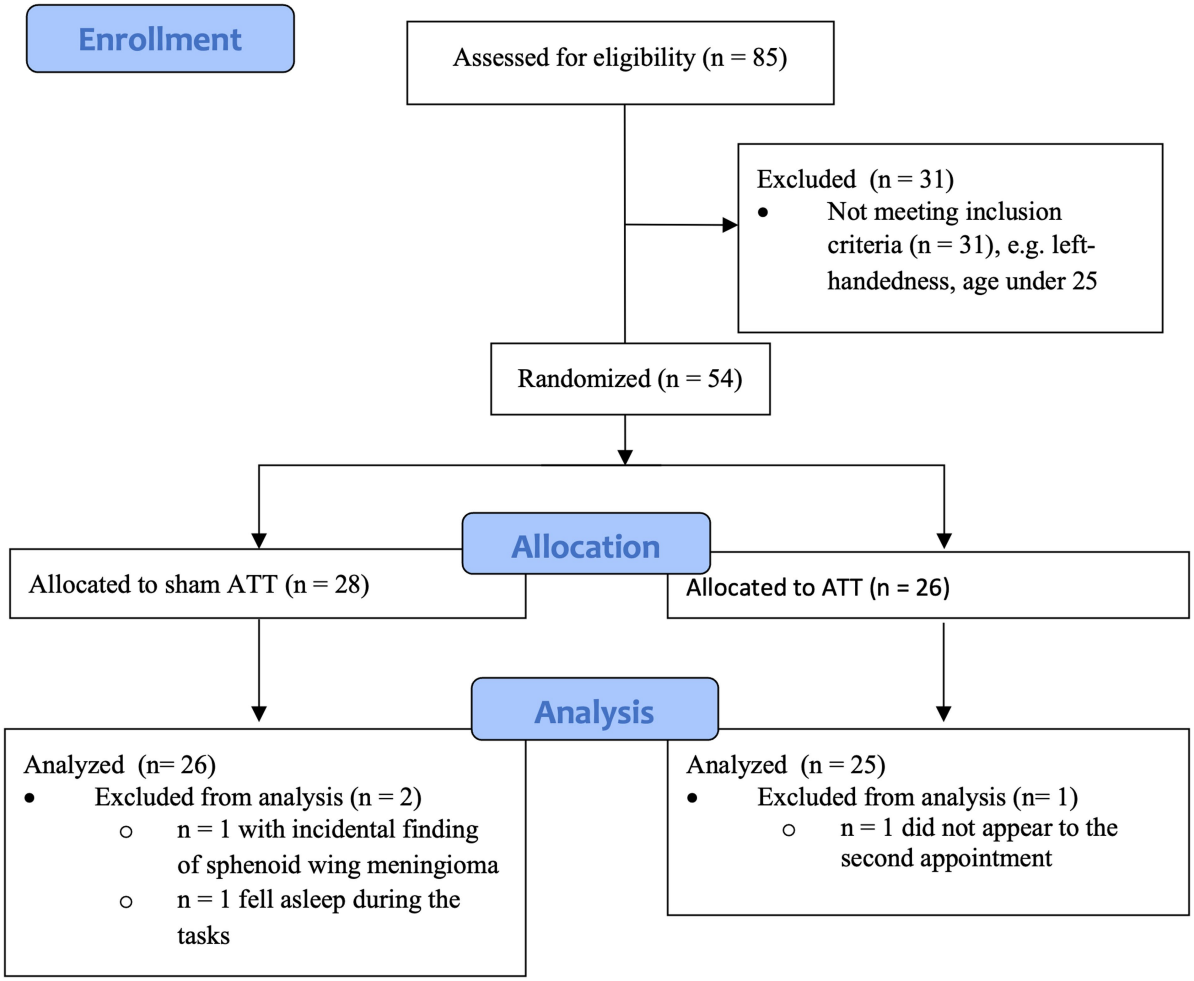
Diagram showing enrollment and allocation of groups.

This study included 35 female and 19 male subjects. Mean age was 33.69 years (SD = 7.67). All subjects finished high school, passed at least 10 years of education, and possessed a secondary educational degree. Three data sets had to be discarded across all tests. One subject was excluded due to a sphenoid wing meningioma observed during the initial MRI scan. One participant fell asleep during multiple behavioral tests and during one fMRI scan. One subject did fail to return for the T1 measurements. This resulted in a final sample size of 51 subjects, with 32 female and 19 male subjects (see Figure 1 for an overview). Mean age was 33.73 years (SD = 7.77).

### Procedure

The study was conducted as a randomized, double-blind, placebo-controlled study.

Subjects were randomly allocated to the ATT group or the sham ATT group using an online randomizer. Both groups followed the same procedures with the exception of the ATT/sham training each group received (see *ATT* for further information). Based on the research of Fan et al. (2005), we assumed a required case number estimate of *N* = 25 per group (calculated using G*Power) to statistically detect differences due to training at the neuronal level in the parietal lobe within a group. This is consistent with a determination of case number sizes in fMRI studies by Thirion et al. (2007), who demonstrated that stable and valid fMRI data can be expected from *N* = 20.

**Figure 2:**
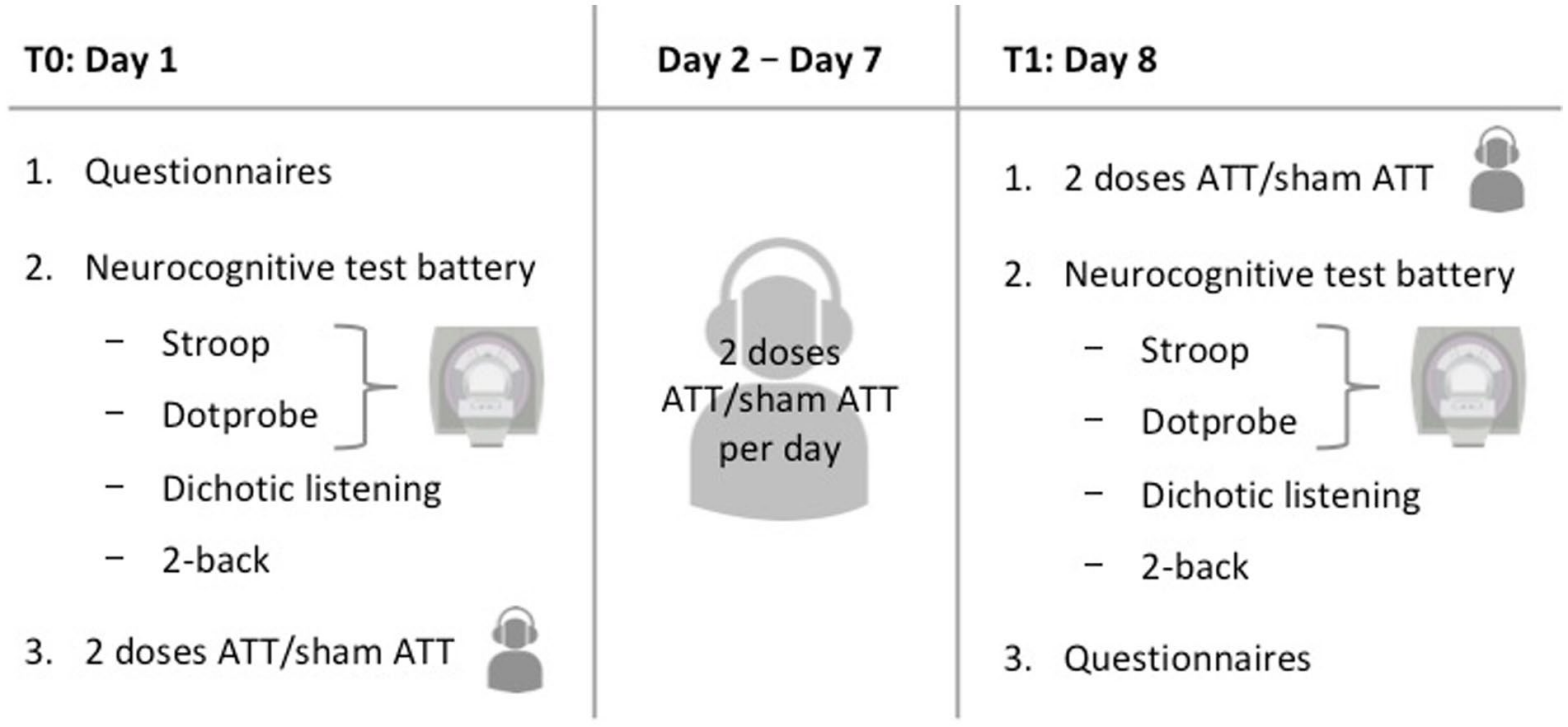
Visual overview of the study procedures.

Participants were required to appear at the study site on two different days with an interval of 1 week between sessions (see Figure 2 for an overview). On day 1, participants were first instructed about the study procedures and signed the informed consent. Upon completion of the questionnaires (see *Questionnaires*), a structured clinical interview for DSM-IV (SCID) was performed by a trained interviewer to confirm the absence of any psychiatric diagnosis. Afterwards, participants performed a test battery consisting of four different tasks (emotional dot probe, Stroop, 2-back and dichotic listening). The first two tasks were conducted inside an fMRI scanner. The session finished with either two sets of ATT or two sets of sham ATT, respectively.

Subsequently, participants were provided with the respective audio file for daily at home training (either ATT or sham ATT). In addition, each participant received a document containing training instructions regarding length and frequency of training. At home training adherence was measured by written self-reports as commonly done with ATT (e.g., Haukaas et al., 2018). Participants were asked to perform ATT/sham ATT twice a day (23 min duration in total) for the next 6 days and to record their training times as well as any omissions, incomplete trainings, or sudden interruptions.

On days 8–1 week after the initial measurements were taken–, participants performed the same tasks as on day 1 again, starting with another two sessions of ATT/sham ATT and finishing with the questionnaires. Finally, a short debriefing concluded the second experimental session. The total experiment time added up to approximately 2:45 h on the first day and 2:20 h on the second day. With two ATT/sham ATT sessions per day the total amount equals 16 sessions. This dosage is comparable to an initial clinical treatment in an MCT-inpatient setting and is an extension of the previous two and four doses used by Barth et al. (2019).

### Attention training technique

The Attention Training Technique (ATT) was applied as described in Barth et al. (2019). The standardized audio file was used according to the ATT instructions and the MCT manual (Wells, 2009).

The ATT audio file starts with a one-minute instructional explanation. A male instructor guides the subject through the listening practice. Six different audio tracks are played simultaneously: crickets chirping, traffic noise, a tolling bell, rushing water, a ticking clock and twittering birds.

A single session of the ATT audio file comprises 12 min including three different stages: selective attention (5 min)–focusing on one auditory stimulus at a time, attentional switching (5 min)–rapid shifting between the auditory stimuli and divided attention (1 min)–focusing on multiple auditory stimuli at once.

The German versions of the ATT/sham ATT, which were used in this study, can be obtained at http://www.metakognitivetherapie.de.

### Sham ATT

Subjects belonging to the sham ATT group followed the same procedure, however, no verbal instructions were given. The participants did not receive any information or instructions regarding the three phases of selective attention, attentional switching and divided attention, but instead solely listened to the six overlapping audio tracks. One session of the sham ATT audio file also lasted 12 min.

### Questionnaires

In order to control for potential confounds concerning depression symptomology and attentional control between both conditions, various questionnaires were used. For attentional control the German versions of the Attentional Control Scale (ACS; Derryberry and Reed, 2002) and the Metacognitive Questionnaire-30 (MCQ; Wells and Cartwright-Hatton, 2004) were implemented. For psychopathological symptomology the Beck Depression Inventory II (BDI-II; Beck et al., 1961, 1996; translated by Kühner et al., 2007), the Borderline Symptom List (BSL-23; Bohus et al., 2009), the Penn State Worry Questionnaire–Past Week (PSWQ-PW, Stöber and Bittencourt, 1998) and the Rumination-Reflection Questionnaire (RRQ; Trapnell and Campbell, 1999; German version by König, 2012) were implemented. Additionally, since this sample comprised only healthy participants, these questionnaires were adopted as a comparison tool for future clinical follow-up studies. Due to study purposes not primary to current research the questionnaire results will not be reported in this paper.

### Experiments

Our goal was to investigate different attentional components (attention bias, attentional flexibility, selective attention), the potential implications of ATT on these as well as potential modality transfer effects of ATT. Therefore, we did not choose ATT-specific tasks, but a range of neurocognitive tests that examined the aforementioned domains as well as specifically near-and far-transfer effects.

The subjects completed a test battery consisting of four different tasks: a Stroop task, an emotional dot probe task, a dichotic listening task and a 2-back task. Each participant received the experimental tasks in the same order.

The Stroop task and the emotional dot probe task were performed in an fMRI setting (see fMRI acquisition). The dichotic listening task and the 2-back task were conducted in a mellow-lighted room at ambient temperature with the participant seated in front of a 19-inch LCD-Screen (Samsung Syncmaster 914n) with Sennheiser HD 558 over-ear headphones.

Presentation® version 18.3 (Neurobehavioral Systems, Inc., Berkeley, CA) was used for programming and presenting the tasks, recording the behavioral data and playing the audio file.

For each individual participant and each task, data sets were excluded when mean reaction time values exceeded three standard deviations of the sample, when subjects failed to follow the task instructions or when subjects showed excessive head movement (> 2 mm i. e. voxel size) in the fMRI scanner. Within each task, single trials were excluded when reaction time values exceeded two standard deviations.

### fMRI acquisition

MR images were acquired using a 3.0-T Siemens MAGNETOM Skyra scanner running Syngo VE11 with a standard 64-channel head coil. An individual high-resolution anatomical scan was acquired using a 6-min T1 weighted magnetization prepared rapid gradient echo imaging sequence with the following parameters: 208 slices, resolution = 1 × 1 × 1 mm, TR = 2,400 ms, TE = 2.13 ms, FOV = 192 × 246 mm, flip angle = 8°. Afterwards, functional data was acquired using gradient simultaneous multislice echoplanar imaging (EPI) T2-sensitive sequences (78 slices, resolution = 2 × 2 × 2 mm, TR = 1,310 ms, TE = 36 ms, FOV = 208 × 208 mm, flip angle = 64°). Another imaging sequence was acquired using non-accelerated T2 weighted sequences for distortion correction (78 slices, resolution 2 × 2 × 2 mm, TR = 7,560 ms, TE 36 ms, FOV 208 mm X 208 mm, flip angle 89°). In addition, 2 non-accelerated spin echo sequences were acquired in anterior–posterior and posterior–anterior directions (78 slices, resolution 2 × 2 X 2 mm, TR = 13,300 ms, TE 118 ms, FOV 208 × 208 mm). Head movements were controlled by monitoring shifts in planes (<2 mm) and rotations (<1°). Head padding was used to minimalize head motion.

After conversion of fMRI images to NIFTI format, all multiband EPI were realigned to the unaccelerated EPI image using FSL mcflirt. This approach allows for motion correction. At the same time, unwarping was conducted with FSL topup using the spin-echo EPI for distortion correction (Andersson et al., 2003). Afterwards coregistration of the mean EPI was performed using the individual high-resolution anatomical scan. Functional images were then normalized to MNI space and smoothed using an 8x8x8mm FWHM Gaussian kernel (Mikl et al., 2008).

Data processing (coregistration, normalization and smoothing) and analysis were conducted using the Matlab based software Statistical Parametric Mapping (SPM) version 12 (Welcome Department of Imaging Neuroscience, University College London).

### fMRI tasks

The emotional dot probe task and the Stroop task were conducted during an fMRI measurement. Thus, participants performed the tasks lying down while looking at a 32-inch Neuro-Nordic-Lab (NNL) monitor *via* a mirror. For each hand, they got a response grip (NNL) with two buttons: one for the thumb and one for the index finger. Instructions of the tasks were given personally prior to the experiments and were presented in written form on screen directly before each task.

The emotional dot probe task lasted 14 min and 32 s (665 volumes) and the Stroop task lasted 13 min and 43 s (628 volumes).

#### Emotional dot probe task

The emotional dot probe task (MacLeod et al., 1986) was used in order to operationalize attentional bias, selective attention performance in the visual domain as well as attentional disengagement from emotional stimuli.

A black cross on a white background served as a fixation point in the center of the screen and was presented during the whole experiment. For each trial, two words were presented simultaneously for 1 s to the left and the right side of the fixation cross. Next, the words disappeared, and an asterisk (a probe) replaced one of the words for 2 s. The subjects were instructed to respond by pressing the button with the index finger that matched the side of the asterisk as fast as possible.

All words shown were either neutral or emotional, with only two combinations allowed: neutral versus neutral word or neutral versus negatively connoted word. To ensure objectivity the ANGST database (Schmidtke et al., 2014) was used to select words. Examples for neutral words used in the task are DOOR (TÜR) and TABLE (TISCH). Emotionally biased words included words associated with grief (e.g., TOMB (GRAB)), pain (e.g., TORTURE (FOLTER)) and fear (e.g., CRASH (ABSTURZ)). The valence of neutral words varied between −0.2 and 0.2 and the valence of emotional words never exceeded−2. The task consisted of 90 trials evenly divided into congruent, incongruent, and neutral trials. Between each trial, a 4 to 8 s inter-trial interval was used with an average of 6 s.

On neutral trials, only neutral words were displayed. On incongruent trials, a neutral as well as a negatively connoted word were displayed and after the words disappeared, the probe appeared on the side of the neutral word (i.e., opposite of the emotional word → attentional disengagement required). On congruent trials, a neutral as well as a negatively connoted word were displayed and after the words disappeared, the probe appeared on the side of the negatively connoted word (→ no attentional disengagement required).

Five data sets had to be discarded. One participant used the thumb instead of the index finger (and respectively, the wrong button) during the second session. One data set exceeded three standard deviations in reaction times (RT). Three data sets were discarded due to excessive head movement in the fMRI scanner. Forty-six data sets remained: *N* = 23 for ATT and *N* = 23 for sham ATT. Regarding behavioral data, the disengaging index (incongruent mean RT–neutral mean RT), bias index (incongruent mean RT–congruent mean RT) and orienting index (neutral mean RT–congruent mean RT) were calculated as described in Salemink et al. (2007).

#### Stroop task

We used the conventional Stroop task without emotional stimuli as another measurement for attentional bias (Stroop, 1935). Our hypothesis implied no significant results due to the absence of emotion in the conventional Stroop task.

Prior to the actual task, subjects completed an exercise block comprising 20 trials. Participants were presented with four different words (RED, YELLOW, GREEN, and BLUE), one at a time. These words were displayed in any of the aforementioned color hues. Subjects had to indicate the color hue of the word rather than the semantic meaning of the word by pressing the respective button as fast as possible: right thumb for blue, right index finger for green, left thumb for red and left index finger for yellow. Two printed hands with accordingly colored thumbs and index fingers were attached to the sides of the monitor as visual indicators.

The task comprised 100 trials. Fifty trials were congruent trials, meaning the hue of the word matched the semantic meaning (e.g., “RED” written in red color), and 50 being incongruent trials, meaning the hue of the word differed from the semantic meaning (e.g., “RED” written in blue color). Each trial lasted 2 s with the colored words being presented for 1 s, while the inter-trial interval measured 4 to 8 s, averaging at 6 s.

After discarding two data sets due to subjects failing to follow task instructions, and one data set due to excessive head movement in the fMRI scanner, 48 data sets were analyzed: *N* = 25 for the ATT group and *N* = 23 for the sham ATT group.

### fMRI data analysis

Data analyzes were performed using the General Linear Model (GLM) implemented in SPM 12. For the emotional dot probe task, the model contained three (incongruent, congruent, neutral) regressors of interest on the single subject level. In addition, six regressors of no interest were included containing the motion parameters. Each boxcar stimulus function was convolved with a canonical hemodynamic response function. The data was then high pass filtered with a cut-off period of 128 s. At a group level the contrast images of each subject representing the analyzed condition were used for random effect analysis. Then, a two-sided t-test was conducted to assess group differences. The threshold for all analyzes was set to *p* ≤ 0.05 family wise error (FWE) corrected for multiple comparisons on cluster level. Peak voxel of significant clusters were localized using automatic anatomical labeling (AAL, Tzourio-Mazoyer et al., 2002). We used *p* = 0.001 as value of *p* per voxel and cluster extend = 0 mm^3^ and report significant results FWE corrected on a cluster level.

Since a healthy study sample was used, only small fMRI effects were expected. Therefore a region-of-interest (ROI) analysis using small volume correction (Poldrack, 2007) was performed based on our *a priori* hypotheses and aforementioned studies about attentional core regions. The left SPL and the ACC are often associated with attention allocation, selective attention and top-down attentional control (Crottaz-Herbette and Menon, 2006; Petersen and Posner, 2012; Shomstein, 2012). Furthermore, the emotional dot probe task and the stroop task are closely associated with the ACC and the SPL (Banich et al., 2000; Klumpp et al., 2012; Thomaes et al., 2012; Price et al., 2014). These regions were therefore used as regions of interest in this study. ROIs were created based on the AAL atlas. A depiction of the ROI mask used in this study can be found in the Supplemental materials.

For the Stroop data, the model contained only two (incongruent, congruent) regressors of interest on the subject level.

### Behavioral tasks

#### Dichotic listening

As another attention bias task, the dichotic listening task was chosen in order to examine potential changes regarding selective auditory attention and a near-transfer effect in the auditory domain. Given the modality congruence between ATT and the dichotic listening task as well as the significant results in the preceding study (Barth et al., 2019), replication of the results was expected. However, this would not be completely in line with MCT theory, since the specific qualities needed for an improvement in this task differ from the ATT-targeted cognitive mechanisms.

As described in Asbjørnsen and Hugdahl (1995), participants had to distinguish between six different auditive stimuli in the form of syllables (ba, da, ga, ka, pa, and ta). These syllables were played as pairs consecutively–one syllable for each ear. All possible syllable combinations were used, amounting to 36 pairs in total. The task was divided into three different sections. At first, subjects had to press the respective key (b, d, g, k, p, and t) on the keyboard according to the syllable they perceived more clearly. During the second and third segment, subjects were told to exclusively shift their attention to the sounds in their right and left ear, respectively.

One data set had to be removed due to missing data, resulting in 50 remaining data sets: *N* = 25 for ATT and *N* = 25 for sham ATT. Analysis were conducted for both ears combined as well as each ear separately. For both ears, the weighted mean of all left and right ear correct reaction times in milliseconds in the forced listening condition constituted the outcome variable. Regarding single-ear testing, the mean of all correct reaction times in milliseconds in the forced listening condition of the respective ear constituted the outcome variable.

#### 2-back

The 2-back task is a standard task used to assess working memory performance and is not an attention bias testing method. Nevertheless, we included it, since working memory processes require similar neurocognitive resources and neural networks. Nevertheless, since ATT does not target working memory performance, it was hypothesized that no significant results will be observed.

Hundred letters were displayed one after another, in random order, in the middle of the computer screen. Once a letter matched the penultimate one, participants had to correspond by pressing “*x*” on the keyboard with their left index finger (target). If they did not match, “*m*” had to be pressed with their right index finger (non-target). There was a 500 ms inter-trial interval between the 1,500 ms lasting displays of each letter.

After the instructor showed a visual on-paper illustration of the task to the participants, 10 exercises trials preceded the actual task to make sure subjects fully understood the task.

Fifty data sets remained after removing one data set due to the subject failing to follow the task instructions: *N* = 25 for ATT and *N* = 25 for sham ATT. The means of correct target and non-target reaction times in milliseconds served as outcome variables.

### Data analysis

Data were analyzed using SPSS Statistics version 23.0 (IBM corp., Amonk, NY). Repeated Analyzes of Variance (ANOVAs) were performed for each task. Since each experimental task tested a distinct capability, we used ATT vs. sham ATT as a factor and the outcome parameters of the individual tasks as dependent variables in order to ascertain an effect in each particular domain and to retest the results of Barth et al. (2019). To rule out any potential confounding effects, age and gender were used as covariates. ACS sum score was also used as a covariate to control for potential confounding effects of baseline differences in attentional control. The inclusion of these covariates did not change the significance of the results, which is why we decided to report the original statistics in this paper. *η*_p_^2^ will be reported as effect size measure for all significant results. No correction method for multiple comparisons (e. g. by Bonferroni-correction) was used to not underpower our partly exploratory study design.

## Results

### Sample characteristics

There were no differences regarding age (*p* = 0.75), sex (*p* = 0.86), ACS total score (*p* = 0.74), BDI sum (*p* = 0.69), BSL sum (*p* = 0.82), MCQ total score (*p* = 0.92), PSWQ-PW sum (*p* = 0.74) and RRQ sum (*p* = 0.847) between the ATT group and the sham ATT group. In addition, there was no difference regarding the frequency of performed ATT/sham ATT at home between both groups (*p* = 0.67; Table 1).

**Table 1:** Data of demographics.

	Condition	*N*	Mean	Standard deviation
Sex (Women/Men)	ATT	25	1.64 (16/9)	0.49
	Sham ATT	26	1.62 (16/10)	0.50
Age	ATT	25	33.36	8.35
	Sham ATT	26	34.08	7.32
Doses of ATT/Sham ATT	ATT	25	14.8	2.24
	Sham ATT	26	15.04	1.71
Total ACS score at T0	ATT	25	60.24	6.92
	Sham ATT	26	59.54	8.03
Total MCQ-30 score at T0	ATT	25	45.48	10.66
	Sham ATT	26	45.15	12.28

### Performance data

#### Emotional dot probe task

We observed significant baseline differences between the ATT and the sham ATT group in the emotional dot probe task when responding to congruent (*p* = 0.02), incongruent (*p* = 0.002) or neutral stimuli (*p* = 0.01). The sham ATT group reacted consistently faster during all trials at T0.

**Figure 3:**
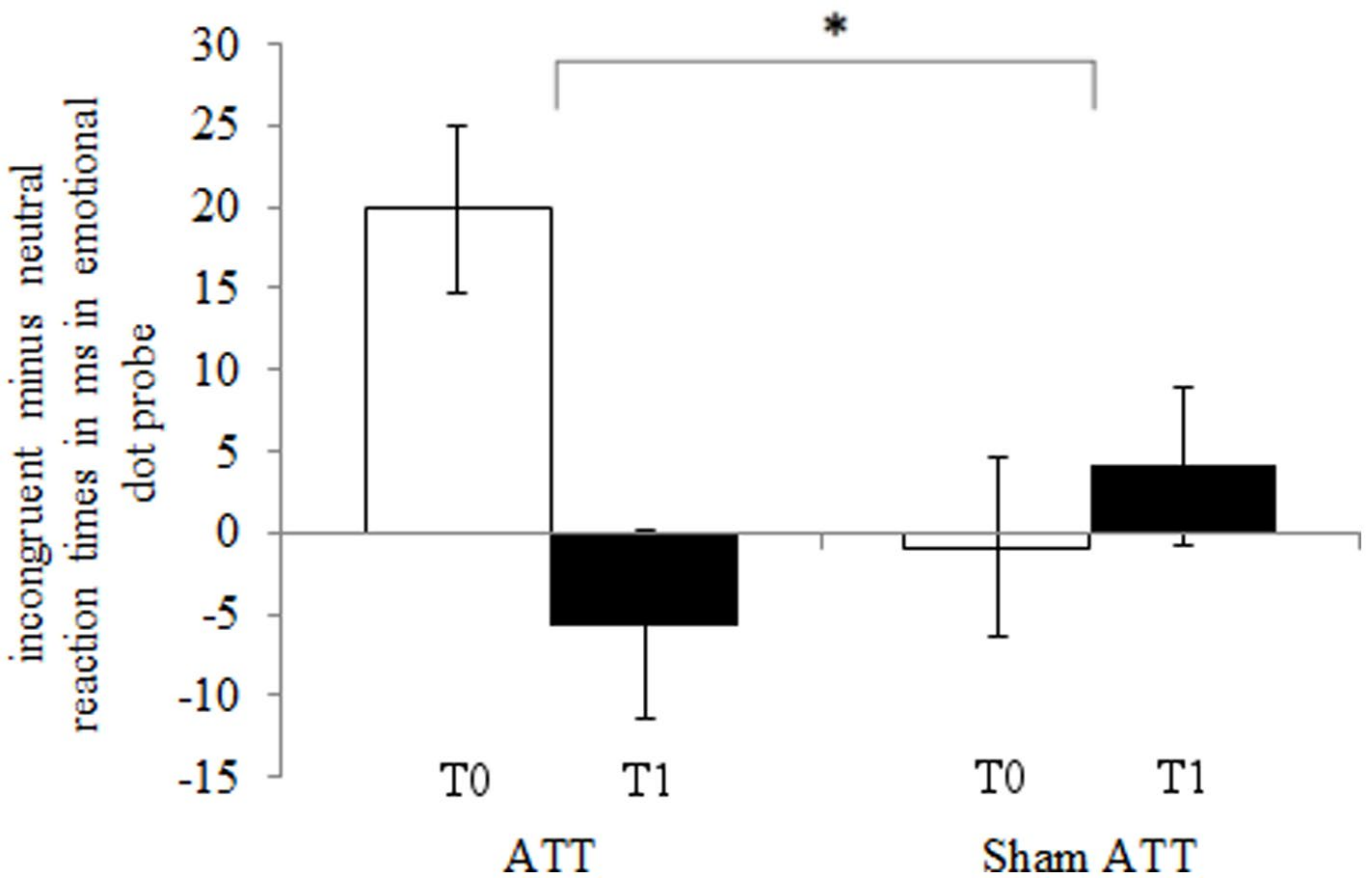
ATT and sham ATT disengaging index (incongruent minus neutral reaction times) during T0 (white) and T1 (black) in the emotional dot probe.

**Figure 4:**
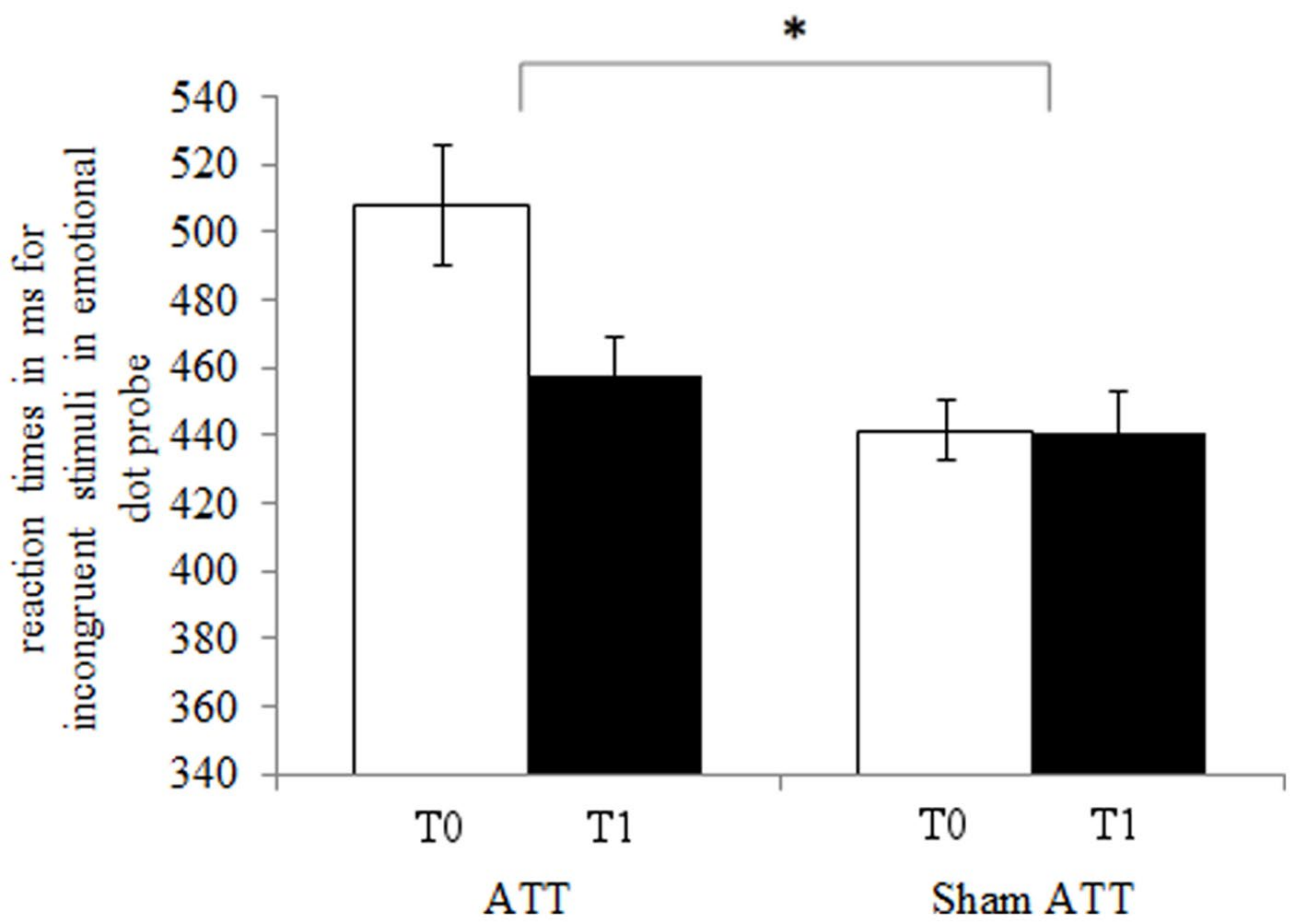
ATT and sham ATT reaction times for incongruent stimuli during T0 (white) and T1 (black) in the emotional dot probe.

The ATT group showed significantly faster disengagement in comparison to the sham ATT group as shown by the disengaging index [incongruent mean RT minus neutral mean RT; *F*(1,44) = 9.25, *p* < 0.01, *η*_p_^2^ = 0.174; see Figure 3] after 1 week. This effect remained significant when an ANCOVA was used with RT from the congruent condition at baseline as covariate and disengagement differences (T1-T0) as dependent variable [*F*(1,43) = 6.36, *p* = 0.015, *η*_p_^2^ = 0.129]. Furthermore, the bias index (incongruent mean RT minus congruent mean RT) showed a non-significant trend in the ATT group in comparison to the sham ATT group [*F*(1,44) = 4.06, *p* = 0.05, *η*_p_^2^ = 0.084]. Please see the Supplemental materials for a corresponding figure of the bias index. No significant ATT-dependent effect was found regarding the orienting index [neutral mean RT minus congruent mean RT; *F*(1,44) = 0.23, *p* = 0.63, *η*_p_^2^ = 0.005]. The ATT group improved significantly when responding to incongruent stimuli in comparison to the sham ATT group [T1-T0; *F*(1,44) = 8.98, *p* < 0.01, *η*_p_^2^ = 0.169; see Figure 4]. No significant change in congruent trial [T1-T0; *F*(1,44) = 3.32, *p* = 0.075, *η*_p_^2^ = 0.07] and neutral trial reaction times [T1-T0; *F*(1,44) = 1.42, *p* = 0.24, *η*_p_^2^ = 0.031] was found between groups.

#### Stroop task

The ATT and the sham ATT group did not significantly differ in the Stroop task regarding reaction times (all *p* values > 0.48) or hits/faults and omitted responses (all *p* values > 0.065) at baseline.

No ATT-dependent effect was found regarding the Stroop task. The incongruent minus congruent reaction times [*F*(1,46) = 0.074, *p* = 0.79] did not show any difference between groups. Furthermore, neither the incongruent reaction times [*F*(1,46) = 0.43, *p* = 0.24] nor the congruent reaction times [*F*(1,46) = 2.03, *p* = 0.16] showed any difference between groups.

#### Dichotic listening task

The ATT and the sham ATT group did not differ in the dichotic listening task regarding reaction times (all *p* values > 0.064) or hits/faults (all *value of p*s > 0.52) at baseline.

First, data for both ears combined was analyzed. No significant reaction time difference was found between groups [T1-T0; *F*(1,48) = 2.46, *p* = 0.12]. However, since a non-significantly larger reduction in reaction times in the ATT group was observed, single-ear analysis was conducted subsequently. Single-ear analysis also revealed no significant reaction time difference between the ATT group and the sham ATT group regarding left or right ear reaction times. However, left ear reaction times showed a non-significant trend [T1-T0; *F*(1,48) = 3.079, *p* = 0.086, *η_p_^2^* = 0.06] for a greater improvement in the ATT group compared to the sham ATT group.

#### 2-back

The ATT and the sham ATT group did not differ in the 2-back task regarding reaction times (all *p* values > 0.58) or hits/faults (all *p* values > 0.34) at baseline.

There was no difference between the ATT group and the sham ATT group regarding correct target counts [*F*(1,48) = 0.616, *p* = 0.436], target reaction times [*F*(1,48) = 0.620, *p* = 0.435] after 1 week of ATT/sham ATT.

### fMRI results

#### Emotional dot probe task

**Figure 5:**
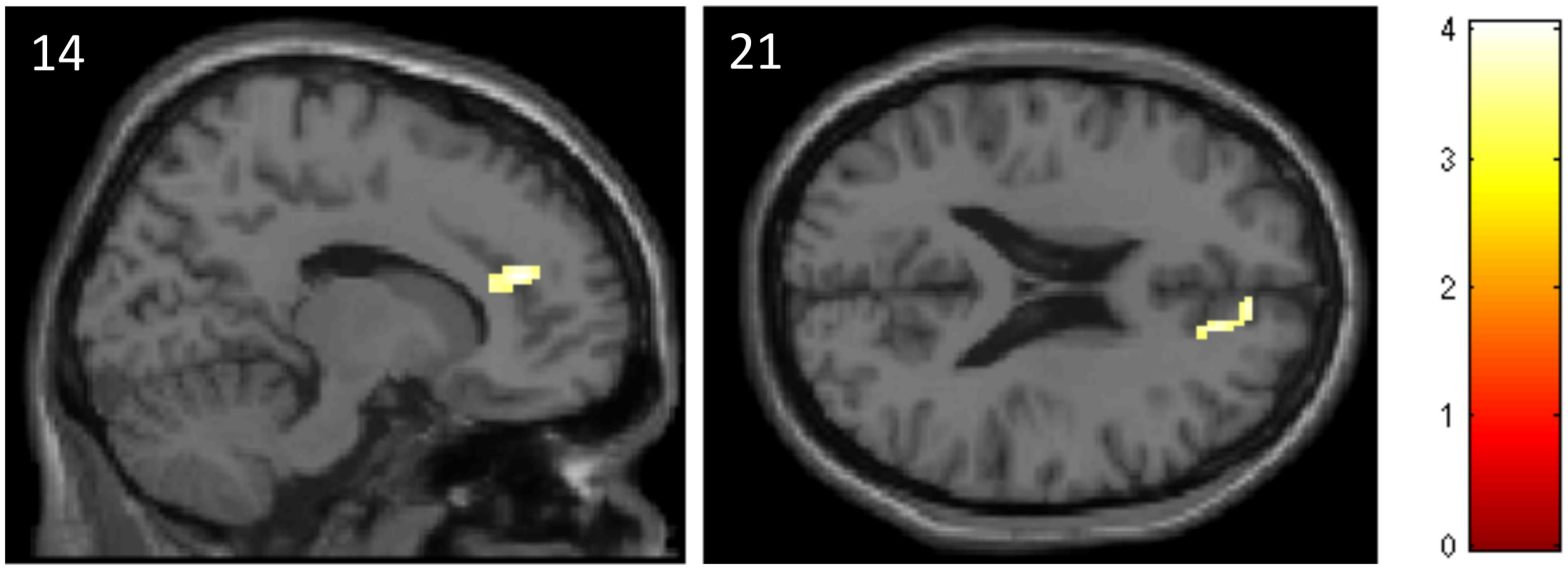
Decreased activation in the anterior cingulate cortex in the ATT group compared to the sham ATT group during incongruent trials of the emotional dot probe task at T1.

The ROI analysis revealed that subjects of the ATT group, compared to the sham ATT group, showed decreased activation in the ACC at T1 when presented with incongruent stimuli (MNI: *x =* 14; *y =* 40; *z =* 21; *p* = 0.045; Table 2, see Figure 5). Other ATT-dependent results regarding congruent or neutral stimuli were not observed. Triple difference analyzes {ATT [T1(incongruent > neutral) > T0(incongruent > neutral)] > sham ATT [T1(incongruent > neutral) > T0(incongruent > neutral)]} did not show any significant effects independent of the direction of testing [e.g., also for (incongruent < neutral) and (T1 < T0)].

**Table 2:** Data and coordinates of the activation changes shown in Figure 5.

location (AAL)	Hemisphere	*x*	*y*	*z*	Cluster size	*p*-value	T-value (peak voxel)
Anterior cingulate cortex	*R*	14	40	20	72 (576 mm^3^)	<0.001	4.04

#### Stroop task

No significant ATT-dependent effects were found comparing the ATT to the sham group or within each group. See supplement for additional information and non-ATT-related effects.

During the “incongruent > congruent” condition significantly elevated levels of activation were found in the left superior parietal lobe and the left precuneus across both groups.

### Correlation between fMRI results and behavioral results

We did not observe any correlations between the fMRI results and the behavioral results (all value of ps greater than 0.06).

## Discussion

The main purpose of this study was to investigate the neural underpinnings of the Attention Training Technique as used in metacognitive psychotherapy in a randomized double-blinded trial using a pre-post fMRI design. In order to examine the various attentional domains affected by ATT, we employed four distinct tasks, with each of them testing partly different attentional domains. Although ATT has shown initial results as a treatment method for various psychiatric conditions, we are still in the fledgling stage regarding the underlying neuronal mechanisms. Hence, two of the four tasks–the Stroop task and the emotional dot probe task–were conducted in an fMRI scanner to further understand the neuronal modus operandi. Both tasks measure attention bias, specifically selective attention in the visual domain, whereas the dichotic listening task tests auditory selective attention and the 2-back task examines working memory performance.

During the emotional dot probe task, subjects in the ATT group showed a significantly larger improvement in attentional disengagement from emotional stimuli–as shown by the disengaging index–in comparison to the sham ATT group. This improvement indicates that the ATT facilitates faster attention allocation toward neutral stimuli, which in turn supports the general concept of the ATT as a method for increasing attentional flexibility. Furthermore, since subjects improved in a visual attentional task while using an auditory training method, these findings display an across-modality-transfer in attentional control.

The fMRI results of the emotional dot probe task showed significantly lower activation in the ACC during incongruent trials at T1 when comparing the ATT to the sham ATT group. Since incongruent trials theoretically require enhanced attentional disengagement from the negatively connoted word, these findings may indicate that the ACC may play a key role in the neuronal mechanisms of attentional disengagement. Furthermore, these results suggest that ATT potentially impacts ACC activity, which in this case might represent the neuronal equivalent of faster attention allocation. In addition, this hints at the CCN as the attentional network, which is influenced by ATT, as the ACC is an integral part of the CCN.

Kowalski et al. (2019) observed decreased connectivity between the ACC, the medial part of the prefrontal cortex and the somatosensory cortex in a group of high-CAS individuals during induced negative thinking and abstract thinking, suggesting a disrupted regulation of threat monitoring. Furthermore, in a paper published, Kowalski et al. (2022) reported a difference in gray matter volume (GMV) in two groups, which differed by their level of CAS–with the high-CAS-group presenting with lower GMV in the left hemisphere dorsal part of the anterior cingulate cortex. Since the ACC is also considered crucial in the neural mechanisms of rumination and worry (Makovac et al., 2020) and this reduction in GMV primarily resides in individuals with high levels of CAS, the observed decreased ACC activity is in line with the behavioral effects of ATT in terms of a reduction in repetitive negative thinking and threat monitoring.

The significantly lower ACC activation observed in the ATT group suggests that frequent ATT training might lead to a functional alteration toward a more flexible attentional steady state, which facilitates attentional reallocation and behaviorally manifests in faster reaction times. In line with this idea, Price et al. (2014) observed a reduction in rostrodorsal ACC activity during a threat-based dot probe task during incongruent trials (attentional disengagement/attention deployment to the neutral side). They also mention a potential “goal-state” of flexible deactivation of parahippocampal regions and attentional reallocation in the ACC, which might be achieved *via* various neurobehavioral training methods such as attention bias modification (ABM) or neuromodulation. With regards to the metacognitive model of disorder these results imply a change in the metacognitive system of the subjects by strengthening or altering metacognitive plans or procedures responsible for attentional disengagement/attentional flexibility. In turn, this portends to the ACC being part of the metacognitive system, possibly a primary factor regarding the storage and application of procedural knowledge, the programs that direct our thought processes (Wells, 2009). In a clinical setting, an enhancement of attentional flexibility, potentially neurally embodied by decreased ACC activation, could directly transfer to the ATT’s ability to modify rumination, threat monitoring and other CAS symptoms.

In patients with major depressive disorder (MDD), the processing of negative emotion is associated with elevated levels of dorsal ACC activation, whereas the processing of positive emotion is associated with decreased levels of dorsal pregenual ACC activation (Groenewold et al., 2013). Furthermore, Pizzagalli (2011) stated that rumination and self-referential processes as part of the MDD can be attenuated by altering resting rACC activity. These findings indicate that ACC activity change might be a potential factor for mitigating depressive symptoms when using the ATT as a stand-alone treatment method.

There were no significant behavioral effects of ATT vs. sham ATT with regard to Stroop task performance. Contrary to the emotional dot probe task the Stroop task also demands executive and inhibitory control (Stroop, 1935). Inhibitory control is needed when the subject is presented with an incongruent trial, meaning a color word, whose semantic meaning does not match its color. Our Stroop task did not possess an emotional component. Thus, the absence of a Stroop performance effect after an intervention aiming to improve attentional disengagement from emotional stimuli is unsurprising.

While we observed the typical Stroop interference effect in form of significantly elevated levels of activation in the left superior parietal lobe and the left precuneus for the “incongruent > congruent” condition across both groups, we found no evidence for an ATT-dependent fMRI effect.

Regarding the dichotic listening task, we found no significant difference in reaction times when comparing the ATT group with the sham ATT group after 1 week of ATT/sham ATT. Single ear analysis showed a trend regarding improvement in reaction times for the left ear in the ATT group in comparison to the sham ATT group.

Since both ATT and the dichotic listening task are linked to the auditory domain, this is a near-transfer task and a transfer of learning from one modality to another is not required. Our findings contradict our hypothesis of a near-transfer task effect, which was observed as an ATT-dependent improvement in both ears in the preceding study by Barth et al. (2019). Hence, these results do not provide any evidence that a one-week ATT intervention does enhance selective attention in the auditory domain. However, the observed trend for left ear reaction times might indicate that there is indeed underlying potential for a global enhancement of selective auditory attention. The reason for a left-side-only trend may be associated with individual differences regarding sensory asymmetries (Porac et al., 1981).

One possible explanation for the disparity in results between this study and the preceding study is the sample group difference. In comparison to the sample group of Barth et al. (2019), which consisted of students only, the sample group of this study is much older and contains a wider variety of occupational and educational backgrounds. Since we see an initial trend for the left ear, a higher degree of statistical power could provide further insight into potential effects for both ears.

The 2-back task, used to assess working memory performance, did not yield any significant ATT-related results. These findings strengthen the implications by Barth et al. (2019). ATT does not seem to impact the working memory domain directly. This was to be expected since the concept of ATT is grounded in enhancing top-down attentional control and improving metacognitive flexibility rather than altering working memory performance.

The overall behavioral data partly replicated the results of Barth et al. (2019), strengthening the general hypothesis that ATT improves attentional disengagement and allocation as well as selective attention, albeit not necessarily in the auditory domain. Functional magnetic resonance data of this study suggests that cerebral regions, which are part of the cognitive control network (CCN), especially the ACC, might be involved in the neuronal processes of the ATT.

### Limitations

This study contains several limitations. Considering the fact that this was a multiple test fMRI-controlled study, the sample size of this study was reasonable. However, studies consisting of a larger sample size could provide a lot more insight and are necessary in order to fully understand neurophysiological ATT effects and further pinpoint ATT manipulated cerebral regions.

One limitation of this study is the between-group baseline difference in the emotional dot-probe task. It is possible that the improvement effect in attentional disengagement is partly driven by this baseline difference. However, there is a time difference effect in the ATT group, which is not present in the sham ATT group. This portends to the existence of a larger improvement in the ATT group than the sham ATT group. Furthermore, regarding the disengaging index the sham ATT group performed worse at T1 than at T0–in contrast to the ATT group, which showed significant improvements. This is indicative of a true intervention effect rather than a baseline driven result. Of course, follow-up studies are needed in order to investigate this further.

Furthermore, only healthy participants were investigated in this study. Thus, deductions regarding any fMRI alterations ATT might have on patients suffering from psychiatric illnesses cannot be directly made. Further studies will have to address this topic using psychiatric patients. First studies are currently conducted in our lab.

Apart from the instruction and the self-report sheet used for measuring adherence, no method was used to control the subjects’ listening times, length and frequencies during the one-week home-training period. Thus, only a base level of 4 doses of ATT for each participant can be fully ensured: 2 at T0 (day 1) and 2 at T1 (day 8). However, the self-report sheets and anecdotal evidence point to a high degree of compliance. Nevertheless, it should not go unmentioned that the recent findings of Heitland et al. (2020) suggest that 4 doses of ATT are in no way inferior to 15 doses of ATT when using a healthy sample group. Not only dosage but also timespan will remain an interesting topic for future follow-up studies, since only a one-week span was looked at in this study whereas application during therapy is usually much longer. Possible investigations could also include long-term fMRI follow-ups in order to observe potential functional cerebral long-term alterations.

Furthermore, we did not include a manipulation check in our study. Participants, however, were neither aware of ATT as a psychotherapeutic treatment method nor of the randomization process and their respective group allocation. The study advertisement document was phrased in general terms and only mentioned that an attention training method will be investigated. In addition, data of the frequency of ATT/sham ATT training did not indicate any motivational differences.

### Conclusion

In conclusion, this study sought to investigate the underlying neuronal mechanisms of the ATT. Although it partly reinforces existing behavioral results on improvements in the visual domain and especially attentional disengagement due to ATT, there was no evidence for an ATT-dependent improvement in auditory selective attention. As one of the first fMRI studies on ATT, it contributes to the localization of cerebral regions involved in the ATT suggesting the CCN, especially the ACC as potential actors when training attentional control and flexibility through ATT. Corresponding to our minor behavioral effects we found a minor ATT-dependent fMRI effect. In summary, this study provides further putative evidence for the behavioral effects of ATT and initial evidence for its corresponding neuronal mechanisms.

Since this is one of the first studies investigating the neurophysiological effects of the ATT, further studies should be conducted for a more thorough understanding of the underlying neural mechanisms regarding metacognition and its attentional elements.

Understanding the “how” behind the Attention Training Technique should lead to a better understanding of attentional control and metacognition in general and could eventually manifest in improved or even more specific treatment. Data replication and research extension to non-healthy participants are definitively essentials on the long path to pinpoint cerebral attentional control regions and to advance metacognitive treatment methods.

## Data availability statement

The raw data supporting the conclusions of this article will be made available by the authors, without undue reservation.

## Ethics statement

The studies involving human participants were reviewed and approved by the ethics committee of the Hannover Medical School. The patients/participants provided their written informed consent to participate in this study.

## Author contributions

CS, IH, and KK designed the experiments. IH performed randomization and allocation of groups. NJ and AB recruited the subjects and collected the data under IH’s and CS’s supervision. NJ, CS, and IH processed and analyzed the data. NJ and IH wrote the first draft of the manuscript. All authors contributed to the article and approved the submitted version.

## Conflict of interest

The authors declare that the research was conducted in the absence of any commercial or financial relationships that could be construed as a potential conflict of interest.

## Publisher’s note

All claims expressed in this article are solely those of the authors and do not necessarily represent those of their affiliated organizations, or those of the publisher, the editors and the reviewers. Any product that may be evaluated in this article, or claim that may be made by its manufacturer, is not guaranteed or endorsed by the publisher.

## References

[ref1] Andersson J. L. R. Skare S. Ashburner J. (2003). How to correct susceptibility distortions in spin-echo echo-planar images: application to diffusion tensor imaging. NeuroImage 20, 870–888. doi: 10.1016/S1053-8119(03)00336-7, PMID: 14568458

[ref2] Armony J. L. Dolan R. J. (2002). Modulation of spatial attention by fear-conditioned stimuli: an event-related fMRI study. Neuropsychologia 40, 817–826. doi: 10.1016/S0028-3932(01)00178-6, PMID: 11900732

[ref3] Asbjørnsen A. E. Hugdahl K. (1995). Attentional effects in dichotic listening. Brain Lang. 49, 189–201. doi: 10.1006/brln.1995.1029, PMID: 7640962

[ref4] Banich M. T. Milham M. P. Atchley R. A. Cohen N. J. Webb A. Wszalek T.. (2000). Prefrontal regions play a predominant role in imposing an attentional “set”: evidence from fMRI. Cogn. Brain Res. 10, 1–9. doi: 10.1016/S0926-6410(00)00015-X, PMID: 10978687

[ref5] Barth V. Heitland I. Kruger T. H. C. Kahl K. G. Sinke C. Winter L. (2019). Shifting instead of drifting-improving attentional performance by means of the attention training technique. Front. Psychol. 10:23. doi: 10.3389/fpsyg.2019.00023, PMID: 30728792 PMC6351493

[ref01] Beck A. T. Steer R. A. Brown G. K. (1996). Manual for the beck depression inventory-II. San Antonio, TX: Psychological Corporation.

[ref6] Beck A. T. Ward C. H. Mendelson M. Mock J. Erbaugh J. (1961). An inventory for measuring depression. Arch. Gen. Psychiatry 4, 561–571. doi: 10.1001/archpsyc.1961.01710120031004, PMID: 13688369

[ref7] Bohus M. Kleindienst N. Limberger M. F. Stieglitz R. D. Domsalla M. Chapman A. L.. (2009). The short version of the borderline symptom list (BSL-23): development and initial data on psychometric properties. Psychopathology 42, 32–39. doi: 10.1159/000173701, PMID: 19023232

[ref8] Botvinick M. M. Carter C. S. Braver T. S. Barch D. M. Cohen J. D. (2001). Conflict monitoring and cognitive control. Psychol. Rev. 108, 624–652. doi: 10.1037/0033-295X.108.3.624, PMID: 11488380

[ref9] Botvinick M. M. Cohen J. D. Carter C. S. (2004). Conflict monitoring and anterior cingulate cortex: an update. Trends Cogn. Sci. 8, 539–546. doi: 10.1016/j.tics.2004.10.003, PMID: 15556023

[ref10] Bush G. Whalen Paul J. Rosen Bruce R. Jenike Michael A. McInerney Sean C. Rauch Scott L. (1998). The counting stroop: an interference task specialized for functional neuroimaging-validation study with functional MRI. Hum. Brain Mapp. 6, 270–282. doi: 10.1002/(SICI)1097-0193(1998)6:4<270::AID-HBM6>3.0.CO;2-0, PMID: 9704265 PMC6873370

[ref11] Callinan S. Johnson D. Wells A. (2014). A randomised controlled study of the effects of the attention training technique on traumatic stress symptoms, emotional attention set shifting and flexibility. Cogn. Ther. Res. 39, 4–13. doi: 10.1007/s10608-014-9634-8

[ref12] Capobianco L. Morris J. A. Wells A. (2018). Worry and rumination: do they prolong physiological and affective recovery from stress? Anxiety Stress Coping 31, 291–303. doi: 10.1080/10615806.2018.1438723, PMID: 29433340

[ref13] Cole M. W. Schneider W. (2007). The cognitive control network: integrated cortical regions with dissociable functions. NeuroImage 37, 343–360. doi: 10.1016/j.neuroimage.2007.03.071, PMID: 17553704

[ref14] Corbetta M. Patel G. Shulman G. L. (2008). The reorienting system of the human brain: from environment to theory of mind. Neuron 58, 306–324. doi: 10.1016/j.neuron.2008.04.017, PMID: 18466742 PMC2441869

[ref15] Crottaz-Herbette S. Menon V. (2006). Where and when the anterior cingulate cortex modulates attentional response: combined fMRI and ERP evidence. J. Cogn. Neurosci. 18, 766–780. doi: 10.1162/jocn.2006.18.5.766, PMID: 16768376

[ref16] Derryberry D. Reed M. A. (2002). Anxiety-related attentional biases and their regulation by attentional control. J. Abnorm. Psychol. 111, 225–236. doi: 10.1037/0021-843X.111.2.225, PMID: 12003445

[ref17] Ehring T. Watkins E. R. (2008). Repetitive negative thinking as a transdiagnostic process. Int. J. Cogn. Ther. 1, 192–205. doi: 10.1521/ijct.2008.1.3.192, PMID: 36794332

[ref18] Fan J. McCandliss B. D. Fossella J. Flombaum J. I. Posner M. I. (2005). The activation of attentional networks. NeuroImage 26, 471–479. doi: 10.1016/j.neuroimage.2005.02.004, PMID: 15907304

[ref19] Fergus T. A. Bardeen J. R. Orcutt H. K. (2012). Attentional control moderates the relationship between activation of the cognitive attentional syndrome and symptoms of psychopathology. Pers. Individ. Dif. 53, 213–217. doi: 10.1016/j.paid.2012.03.017

[ref20] Fergus T. A. Wheless N. E. (2018). The attention training technique causally reduces self-focus following worry provocation and reduces cognitive anxiety among self-focused individuals. J. Behav. Ther. Exp. Psychiatry 61, 66–71. doi: 10.1016/j.jbtep.2018.06.006, PMID: 29929054

[ref21] Fergus T. A. Wheless N. E. Wright L. C. (2014). The attention training technique, self-focused attention, and anxiety: a laboratory-based component study. Behav. Res. Ther. 61, 150–155. doi: 10.1016/j.brat.2014.08.007, PMID: 25213665

[ref22] Flavell J. H. (1979). Metacognition and cognitive monitoring: a new area of cognitive-developmental inquiry. Am. Psychol. 34, 906–911. doi: 10.1037/0003-066X.34.10.906

[ref23] Groenewold N. A. Opmeer E. M. de Jonge P. Aleman A. Costafreda S. G. (2013). Emotional valence modulates brain functional abnormalities in depression: evidence from a meta-analysis of fMRI studies. Neurosci. Biobehav. Rev. 37, 152–163. doi: 10.1016/j.neubiorev.2012.11.015, PMID: 23206667

[ref24] Gronwall D. M. A. (1977). Paced auditory serial addition task: a measure of recovery from concussion. Percept. Mot. Skills 44, 367–373. doi: 10.2466/pms.1977.44.2.367, PMID: 866038

[ref25] Hanlon F. M. Dodd A. B. Ling J. M. Bustillo J. R. Abbott C. C. Mayer A. R. (2017). From behavioral facilitation to inhibition: the neuronal correlates of the orienting and reorienting of auditory attention. Front. Hum. Neurosci. 11, 1–10. doi: 10.3389/fnhum.2017.00293, PMID: 28634448 PMC5459904

[ref26] Haukaas R. B. Gjerde I. B. Varting G. Hallan H. E. Solem S. (2018). A randomized controlled trial comparing the attention training technique and mindful self-compassion for students with symptoms of depression and anxiety. Front. Psychol. 9:827. doi: 10.3389/fpsyg.2018.00827, PMID: 29887823 PMC5982936

[ref27] Heitland I. Barth V. Winter L. Jahn N. Burak A. Sinke C.. (2020). One step ahead–attention control capabilities at baseline are associated with the effectiveness of the attention training technique. Front. Psychol. 11:401. doi: 10.3389/fpsyg.2020.00401, PMID: 32296360 PMC7136490

[ref28] Kitchener K. S. (1983). Cognition, metacognition, and epistemic cognition: a three-level model of cognitive processing. Hum. Dev. 26, 222–232. doi: 10.1159/000272885

[ref29] Klumpp H. Angstadt M. Luan Phan K. (2012). Shifting the focus of attention modulates amygdala and anterior cingulate cortex reactivity to emotional faces. Neurosci. Lett. 514, 210–213. doi: 10.1016/j.neulet.2012.03.003, PMID: 22425719 PMC3598557

[ref30] Knowles M. M. Foden P. El-Deredy W. Wells A. (2016). A systematic review of efficacy of the attention training technique in clinical and nonclinical samples. J. Clin. Psychol. 72, 999–1025. doi: 10.1002/jclp.22312, PMID: 27129094

[ref31] Knowles M. M. Wells A. (2018). Single dose of the attention training technique increases resting alpha and beta-oscillations in frontoparietal brain networks: a randomized controlled comparison. Front. Psychol. 9, 1–9. doi: 10.3389/fpsyg.2018.01768, PMID: 30294294 PMC6158576

[ref32] König D. (2012). *Deutsche Version der Skala Rumination aus dem Rumination-Reflection*. Questionnaire (RRQ).

[ref33] Kowalski J. Wierzba M. Wypych M. Marchewka A. Dragan M. (2020). Effects of attention training technique on brain function in high-and low-cognitive-attentional syndrome individuals: regional dynamics before, during, and after a single session of ATT. Behav. Res. Ther. 132:103693. doi: 10.1016/j.brat.2020.103693, PMID: 32688045

[ref34] Kowalski J. Wypych M. Marchewka A. Dragan M. (2019). Neural correlates of cognitive-attentional syndrome: an fMRI study on repetitive negative thinking induction and resting state functional connectivity. Front. Psychol. 10:648. doi: 10.3389/fpsyg.2019.00648, PMID: 30971987 PMC6443848

[ref35] Kowalski J. Wypych M. Marchewka A. Dragan M. (2022). Brain structural correlates of cognitive-attentional syndrome-a voxel-based morphometry study. Brain Imaging Behav. 16, 1914–1918. doi: 10.1007/s11682-022-00649-2. Epub 2022 Mar 10, PMID: 35266100

[ref36] Kühner C. Bürger C. Keller F. Hautzinger M. (2007). Reliabilität und validität des revidierten Beck-Depressionsinventars (BDI-II). Nervenarzt 78, 651–656. doi: 10.1007/s00115-006-2098-7, PMID: 16832698

[ref37] Lee A. K. C. Larson E. Maddox R. K. Shinn-Cunningham B. G. (2014). Using neuroimaging to understand the cortical mechanisms of auditory selective attention. Hear. Res. 307, 111–120. doi: 10.1016/j.heares.2013.06.010, PMID: 23850664 PMC3844039

[ref38] MacLeod C. Mathews A. Tata P. (1986). Attentional bias in emotional disorders. J. Abnorm. Psychol. 95, 15–20. doi: 10.1037/0021-843X.95.1.15, PMID: 3700842

[ref39] Makovac E. Fagioli S. Rae C. L. Critchley H. D. Ottaviani C. (2020). Can’t get it off my brain: meta-analysis of neuroimaging studies on perseverative cognition. Psychiatry Res. Neuroimaging 295:111020. doi: 10.1016/j.pscychresns.2019.111020, PMID: 31790922

[ref40] Maxwell S. E. Lau M. Y. Howard G. S. (2015). Is psychology suffering from a replication crisis?: what does “failure to replicate” really mean? Am. Psychol. 70, 487–498. doi: 10.1037/a0039400, PMID: 26348332

[ref41] McEvoy P. M. Graville R. Hayes S. Kane R. T. Foster J. K. (2017). Mechanisms of change during attention training and mindfulness in high trait-anxious individuals: a randomized controlled study. Behav. Ther. 48, 678–694. doi: 10.1016/j.beth.2017.04.001, PMID: 28711117

[ref42] Mikl M. Mareček R. Hluštík P. Pavlicová M. Drastich A. Chlebus P.. (2008). Effects of spatial smoothing on fMRI group inferences. Magn. Reson. Imaging 26, 490–503. doi: 10.1016/j.mri.2007.08.006, PMID: 18060720

[ref43] Milham M. P. Banich M. T. Webb A. Barad V. Cohen N. J. Wszalek T.. (2001). The relative involvement of anterior cingulate and prefrontal cortex in attentional control depends on nature of conflict. Cogn. Brain Res. 12, 467–473. doi: 10.1016/S0926-6410(01)00076-3, PMID: 11689307

[ref44] Moisala M. Salmela V. Salo E. Carlson S. Vuontela V. Salonen O.. (2015). Brain activity during divided and selective attention to auditory and visual sentence comprehension tasks. Front. Hum. Neurosci. 9, 1–15. doi: 10.3389/fnhum.2015.00086, PMID: 25745395 PMC4333810

[ref45] Murray J. Scott H. Connolly C. Wells A. (2018). The attention training technique improves Children’s ability to delay gratification: a controlled comparison with progressive relaxation. Behav. Res. Ther. 104, 1–6. doi: 10.1016/j.brat.2018.02.003, PMID: 29471185

[ref46] Myhr P. Hursti T. Emanuelsson K. Löfgren E. Hjemdal O. (2019). Can the attention training technique reduce stress in students? A controlled study of stress appraisals and meta-worry. Front. Psychol. 10:1532. doi: 10.3389/fpsyg.2019.01532, PMID: 31354569 PMC6635479

[ref47] Nassif Y. Wells A. (2014). Attention training reduces intrusive thoughts cued by a narrative of stressful life events: a controlled study. J. Clin. Psychol. 70, 510–517. doi: 10.1002/jclp.22047, PMID: 24114746

[ref48] Nolen-Hoeksema S. (1991). Responses to depression and their effects on the duration of depressive episodes. J. Abnorm. Psychol. 100, 569–582. doi: 10.1037/0021-843X.100.4.569, PMID: 1757671

[ref49] Petersen S. E. Posner M. I. (2012). The attention system of the human brain: 20 years after. Annu. Rev. Neurosci. 35, 73–89. doi: 10.1146/annurev-neuro-062111-150525, PMID: 22524787 PMC3413263

[ref50] Pizzagalli D. A. (2011). Frontocingulate dysfunction in depression: toward biomarkers of treatment response. Neuropsychopharmacology 36, 183–206. doi: 10.1038/npp.2010.166, PMID: 20861828 PMC3036952

[ref51] Poldrack R. A. (2007). Region of interest analysis for fMRI. Soc. Cogn. Affect. Neurosci. 2, 67–70. doi: 10.1093/scan/nsm006, PMID: 18985121 PMC2555436

[ref52] Porac C. Coren S. Porac C. Coren S. (1981). Lateral Preferences and Human Behavior. New York: Springer.

[ref53] Pourtois G. Schwartz S. Seghier M. L. Lazeyras F. Vuilleumier P. (2006). Neural systems for orienting attention to the location of threat signals: an event-related fMRI study. NeuroImage 31, 920–933. doi: 10.1016/j.neuroimage.2005.12.034, PMID: 16487729

[ref54] Price R. B. Siegle G. J. Silk J. S. Ladouceur C. D. McFarland A. Dahl R. E.. (2014). Looking under the hood of the dot-probe task: an fmri study in anxious youth. Depress. Anxiety 31, 178–187. doi: 10.1002/da.22255, PMID: 24578016 PMC3992818

[ref55] Rosenbaum D. Maier M. J. Hudak J. Metzger F. G. Wells A. Fallgatter A. J.. (2018). Neurophysiological correlates of the attention training technique: a component study. Neuroimage Clin. 19, 1018–1024. doi: 10.1016/j.nicl.2018.06.021, PMID: 30003039 PMC6039840

[ref56] Salemink E. van den Hout M. A. Kindt M. (2007). Selective attention and threat: quick orienting versus slow disengagement and two versions of the dot probe task. Behav. Res. Ther. 45, 607–615. doi: 10.1016/j.brat.2006.04.004, PMID: 16769035

[ref57] Salmi J. Rinne T. Degerman A. Salonen O. Alho K. (2007). Orienting and maintenance of spatial attention in audition and vision: multimodal and modality-specific brain activations. Brain Struct. Funct. 212, 181–194. doi: 10.1007/s00429-007-0152-2, PMID: 17717689

[ref58] Schmidtke D. S. Schröder T. Jacobs A. M. Conrad M. (2014). ANGST: affective norms for German sentiment terms, derived from the affective norms for English words. Behav. Res. Methods 46, 1108–1118. doi: 10.3758/s13428-013-0426-y, PMID: 24415407

[ref59] Sharpe L. Nicholson Perry K. Rogers P. Dear B. F. Nicholas M. K. Refshauge K. (2010). A comparison of the effect of attention training and relaxation on responses to pain. Pain 150, 469–476. doi: 10.1016/j.pain.2010.05.027, PMID: 20619540

[ref60] Shomstein S. (2012). Cognitive functions of the posterior parietal cortex: top-down and bottom-up attentional control. Front. Integr. Neurosci. 6, 1–7. doi: 10.3389/fnint.2012.00038, PMID: 22783174 PMC3389368

[ref61] Shomstein S. Yantis S. (2006). Parietal cortex mediates voluntary control of spatial and nonspatial auditory attention. J. Neurosci. 26, 435–439. doi: 10.1523/JNEUROSCI.4408-05.2006, PMID: 16407540 PMC6674402

[ref62] Siegle G. J. Ghinassi F. Thase M. E. (2007). Neurobehavioral therapies in the 21st century: summary of an emerging field and an extended example of cognitive control training for depression. Cogn. Ther. Res. 31, 235–262. doi: 10.1007/s10608-006-9118-6

[ref63] Slagter H. A. Giesbrecht B. Kok A. Weissman D. H. Kenemans J. L. Woldorff M. G.. (2007). fMRI evidence for both generalized and specialized components of attentional control. Brain Res. 1177, 90–102. doi: 10.1016/j.brainres.2007.07.097, PMID: 17916338 PMC2710450

[ref64] Stewart K. E. Antony M. M. Koerner N. (2021). A randomized experimental analysis of the attention training technique: effects on worry and relevant processes in individuals with probable generalized anxiety disorder. Behav. Res. Ther. 141:103863. doi: 10.1016/j.brat.2021.103863, PMID: 33872957

[ref65] Stöber J. Bittencourt J. (1998). Weekly assessment of worry: an adaptation of the Penn State worry questionnaire for monitoring changes during treatment. Behav. Res. Ther. 36, 645–656. doi: 10.1016/S0005-7967(98)00031-X, PMID: 9648338

[ref66] Stroop J. R. (1935). Studies of interference in serial verbal reactions. J. Exp. Psychol. 18, 643–662. doi: 10.1037/h0054651, PMID: 33211511

[ref67] Thirion B. Pinel P. Mériaux A. Roche A. Dehaene S. Poline J.-B. (2007). Analysis of a large fMRI cohort: statistical and methodological issues for group analyses. NeuroImage 35, 105–120. doi: 10.1016/j.neuroimage.2006.11.054, PMID: 17239619

[ref68] Thomaes K. Dorrepaal E. Draijer N. De Ruiter M. Elzinga B. Van Balkom A.. (2012). Treatment effects on insular and anterior cingulate cortex activation during classic and emotional Stroop interference in child abuse-related complex post-traumatic stress disorder. Psychol. Med. 42, 2337–2349. doi: 10.1017/S0033291712000499, PMID: 22436595

[ref69] Trapnell P. D. Campbell J. D. (1999). Private self-consciousness and the five-factor model of personality: distinguishing rumination from reflection. J. Pers. Soc. Psychol. 76, 284–304. doi: 10.1037/0022-3514.76.2.284, PMID: 10074710

[ref70] Trick L. Watkins E. R. Henley W. Gandhi M. M. Dickens C. (2019). Perseverative negative thinking predicts depression in people with acute coronary syndrome. Gen. Hosp. Psychiatry 61, 16–25. doi: 10.1016/j.genhosppsych.2019.06.012, PMID: 31733604

[ref71] Tzourio-Mazoyer N. Landeau B. Papathanassiou D. Crivello F. Etard O. Delcroix N.. (2002). Automated anatomical labeling of activations in SPM using a macroscopic anatomical parcellation of the MNI MRI single-subject brain. NeuroImage 15, 273–289. doi: 10.1006/nimg.2001.0978, PMID: 11771995

[ref72] Van Veen V. Carter C. S. (2002). The anterior cingulate as a conflict monitor: FMRI and ERP studies. Physiol. Behav. 77, 477–482. doi: 10.1016/S0031-9384(02)00930-7, PMID: 12526986

[ref73] Vossel S. Geng J. J. Fink G. R. (2014). Dorsal and ventral attention systems: distinct neural circuits but collaborative roles. Neuroscientist 20, 150–159. doi: 10.1177/1073858413494269, PMID: 23835449 PMC4107817

[ref74] Wells A. (1990). Panic disorder in association with relaxation induced anxiety: an attentional training approach to treatment. Behav. Ther. 21, 273–280. doi: 10.1016/S0005-7894(05)80330-2, PMID: 19059753

[ref75] Wells A. (2009). Metacognitive Therapy for Anxiety and Depression. New York: Guilford Wells

[ref76] Wells A. (2019). Breaking the cybernetic code: understanding and treating the human metacognitive control system to enhance mental health. Front. Psychol. 10:2621. doi: 10.3389/fpsyg.2019.02621, PMID: 31920769 PMC6920120

[ref77] Wells A. Cartwright-Hatton S. (2004). A short form of the metacognitions questionnaire: properties of the MCQ-30. Behav. Res. Ther. 42, 385–396. doi: 10.1016/S0005-7967(03)00147-5, PMID: 14998733

[ref78] Wells A. Matthews G. (1996). Modelling cognition in emotional disorder: the S-REF model. Behav. Res. Ther. 34, 881–888. doi: 10.1016/S0005-7967(96)00050-2, PMID: 8990539

