# Supplementary material for: Neural correlates of the attention training technique as used in metacognitive therapy – A randomized sham-controlled fMRI study in healthy volunteers
Source: Front Psychol. 2023 Mar 13;14:1084022. doi: 10.3389/fpsyg.2023.1084022 (PMC10040584; doi:10.3389/fpsyg.2023.1084022)
Supplement: Supplementary file 1 [file Table_1.DOCX]

Supplementary material

**1. fMRI data analysis**


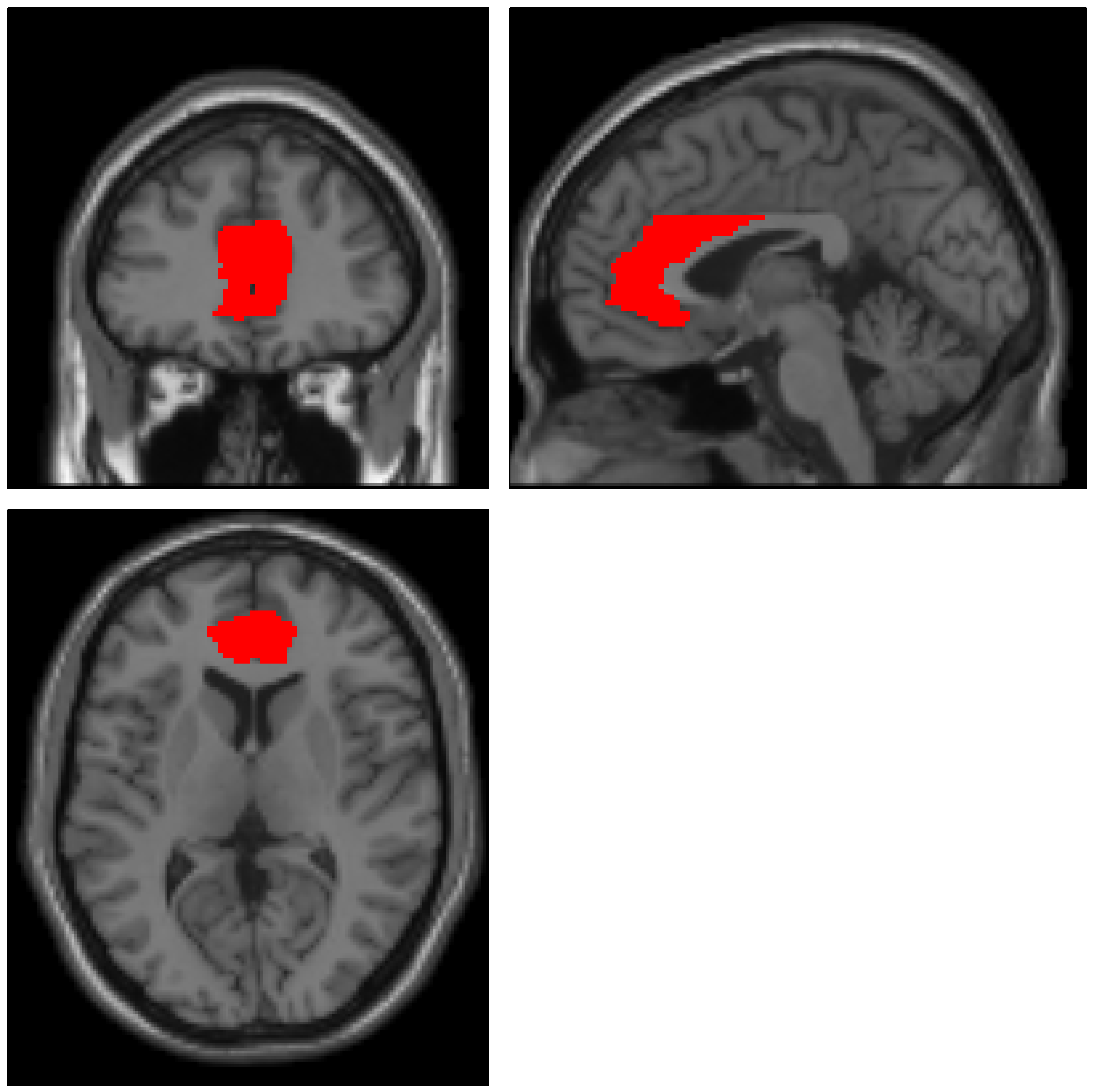


**Figure 1**. Depiction of ROI mask of the anterior cingulate cortex used in the ROI analysis


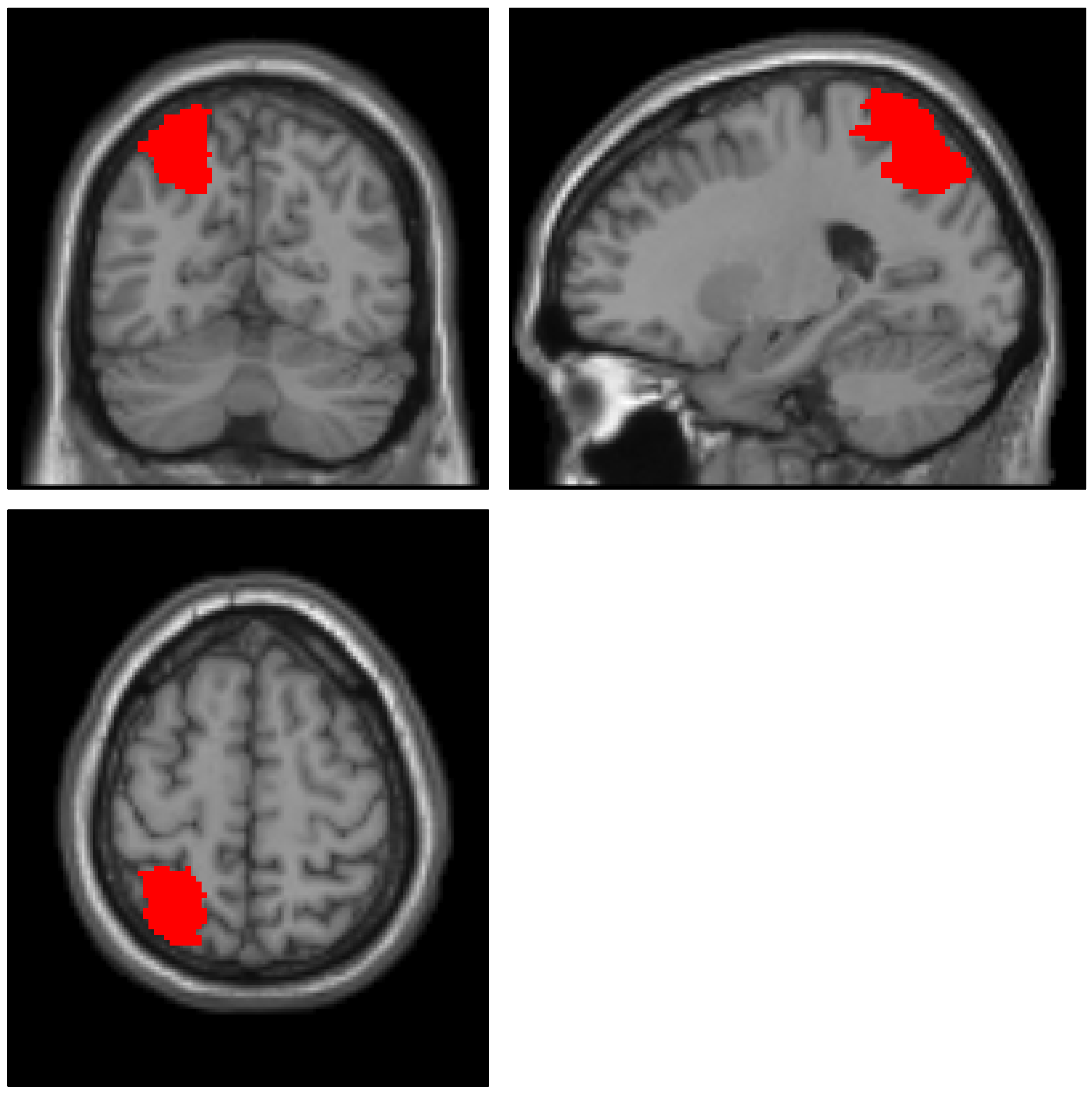


**Figure 2**. Depiction of ROI mask of the superior parietal lobe used in the ROI analysis

**2. Results**

**Table 1**: Individual difference measures at T0

|  | **Condition** | **N** | **Mean** | **Standard deviation** |
| --- | --- | --- | --- | --- |
| Reaction times for correct targets at T0 in the 2back task | ATT | 25 | 681.04 | 161.08 |
|  | Sham ATT | 26 | 648.17 | 176.76 |
| Weighted mean of left an right correct reaction times at T0 in the dichotic listening task | ATT | 25 | 1156.06 | 155.33 |
|  | Sham ATT | 25 | 1086.38 | 106.68 |
| Mean of reaction times for congruent stimuli at T0 in the emotional dot probe task | ATT | 25 | 491.62 | 66.44 |
|  | Sham ATT | 26 | 448.96 | 54.27 |
| Mean of reaction times for incongruent stimuli at T0 in the emotional dot probe task | ATT | 25 | 513.19 | 83.87 |
|  | Sham ATT | 26 | 445.67 | 47.07 |
| Mean of reaction times for neutral stimuli at T0 in the emotional dot probe task | ATT | 25 | 491.28 | 71.94 |
|  | Sham ATT | 26 | 446.88 | 43.91 |
| Bias index at T0 in the emotional dot probe task | ATT | 25 | 21.57 | 29.99 |
|  | Sham ATT | 26 | -3.28 | 27.16 |
| Orienting index at T0 in the emotional dot probe task | ATT | 25 | -0.34 | 17.84 |
|  | Sham ATT | 26 | -2.07 | 26.54 |
| Disengaging index at T0 in the emotional dot probe task | ATT | 25 | 21.91 | 25.48 |
|  | Sham ATT | 26 | -1.21 | 25.04 |
| Mean of reaction times for congruent stimuli at T0 in the Stroop task | ATT | 25 | 817.10 | 162.31 |
|  | Sham ATT | 26 | 788.42 | 126.29 |
| Mean of reaction times for incongruent stimuli at T0 in the Stroop task | ATT | 25 | 891.17 | 195.97 |
|  | Sham ATT | 26 | 858.48 | 182.39 |
| Total BDI score at T0 | ATT | 25 | 4.32 | 6.14 |
|  | Sham ATT | 26 | 5.04 | 6.61 |
| Total BSL score at T0 | ATT | 25 | 4.08 | 3.71 |
|  | Sham ATT | 26 | 4.39 | 5.42 |
| BSL mean at T0 | ATT | 25 | 0.18 | 0.16 |
|  | Sham ATT | 26 | 0.19 | 0.24 |
| Total PSWQ score at T0 | ATT | 25 | 23.92 | 16.32 |
|  | Sham ATT | 26 | 25.39 | 15.40 |
| Total RRQ score at T0 | ATT | 25 | 32.32 | 8.63 |
|  | Sham ATT | 26 | 32.85 | 10.60 |

**Table 2**: Individual difference measures at T1

|  | **Condition** | **N** | **Mean** | **Standard deviation** |
| --- | --- | --- | --- | --- |
| Reaction times for correct targets at T1 in the 2back task | ATT | 25 | 631.46 | 169.13 |
|  | Sham ATT | 26 | 621.08 | 148.04 |
| Weighted mean of left an right correct reaction times at T1 in the dichotic listening task | ATT | 25 | 1087.32 | 141.28 |
|  | Sham ATT | 26 | 1060.43 | 107.75 |
| Mean of reaction times for congruent stimuli at T1 in the emotional dot probe task | ATT | 25 | 467.87 | 85.45 |
|  | Sham ATT | 26 | 439.36 | 61.01 |
| Mean of reaction times for incongruent stimuli at T1 in the emotional dot probe task | ATT | 25 | 467.75 | 73.33 |
|  | Sham ATT | 26 | 443.92 | 58.28 |
| Mean of reaction times for neutral stimuli at T1 in the emotional dot probe task | ATT | 25 | 475.79 | 83.01 |
|  | Sham ATT | 26 | 439.80 | 56.41 |
| Bias index at T1 in the emotional dot probe task | ATT | 25 | -0.12 | 35.14 |
|  | Sham ATT | 26 | 4.56 | 32.88 |
| Orienting index at T1 in the emotional dot probe task | ATT | 25 | 7.91 | 21.06 |
|  | Sham ATT | 26 | 0.44 | 25.02 |
| Disengaging index at T1 in the emotional dot probe task | ATT | 25 | -8.04 | 31.97 |
|  | Sham ATT | 26 | 4.12 | 22.24 |
| Mean of reaction times for congruent stimuli at T1 in the Stroop task | ATT | 25 | 768.78 | 119.72 |
|  | Sham ATT | 26 | 770.37 | 119.66 |
| Mean of reaction times for incongruent stimuli at T1 in the Stroop task | ATT | 25 | 823.11 | 151.82 |
|  | Sham ATT | 26 | 848.83 | 150.97 |
| Total BDI score at T1 | ATT | 25 | 2.72 | 5.03 |
|  | Sham ATT | 26 | 4 | 7.15 |
| Total BSL score at T1 | ATT | 25 | 2.44 | 4.47 |
|  | Sham ATT | 26 | 3.35 | 5.08 |
| BSL mean at T1 | ATT | 24 | 0.11 | 0.20 |
|  | Sham ATT | 26 | 0.15 | 0.22 |
| Total PSWQ score at T1 | ATT | 25 | 22.2 | 15.79 |
|  | Sham ATT | 26 | 22.62 | 18.24 |
| Total RRQ score at T1 | ATT | 25 | 30.24 | 11.56 |
|  | Sham ATT | 26 | 30.39 | 10.26 |

*2.1 Behavioral results*

2.1.1 Emotional dot probe


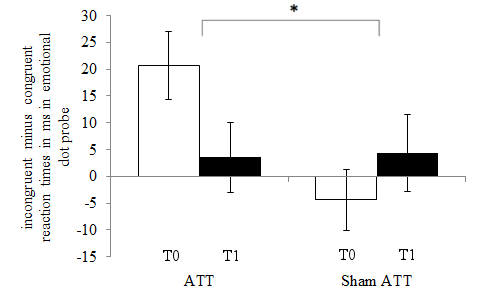


**Figure 2**. ATT and sham ATT bias index (incongruent minus congruent reaction times) during T0 (white) and T1 (black) in the emotional dot probe

*2.2 fMRI results*

2.2.1 Emotional dot probe


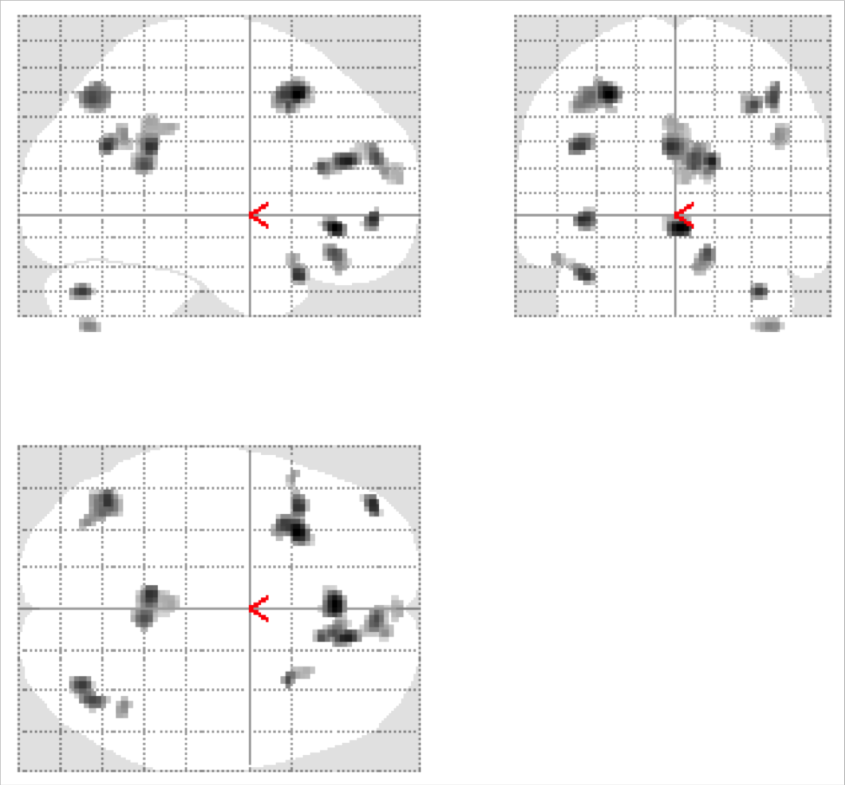


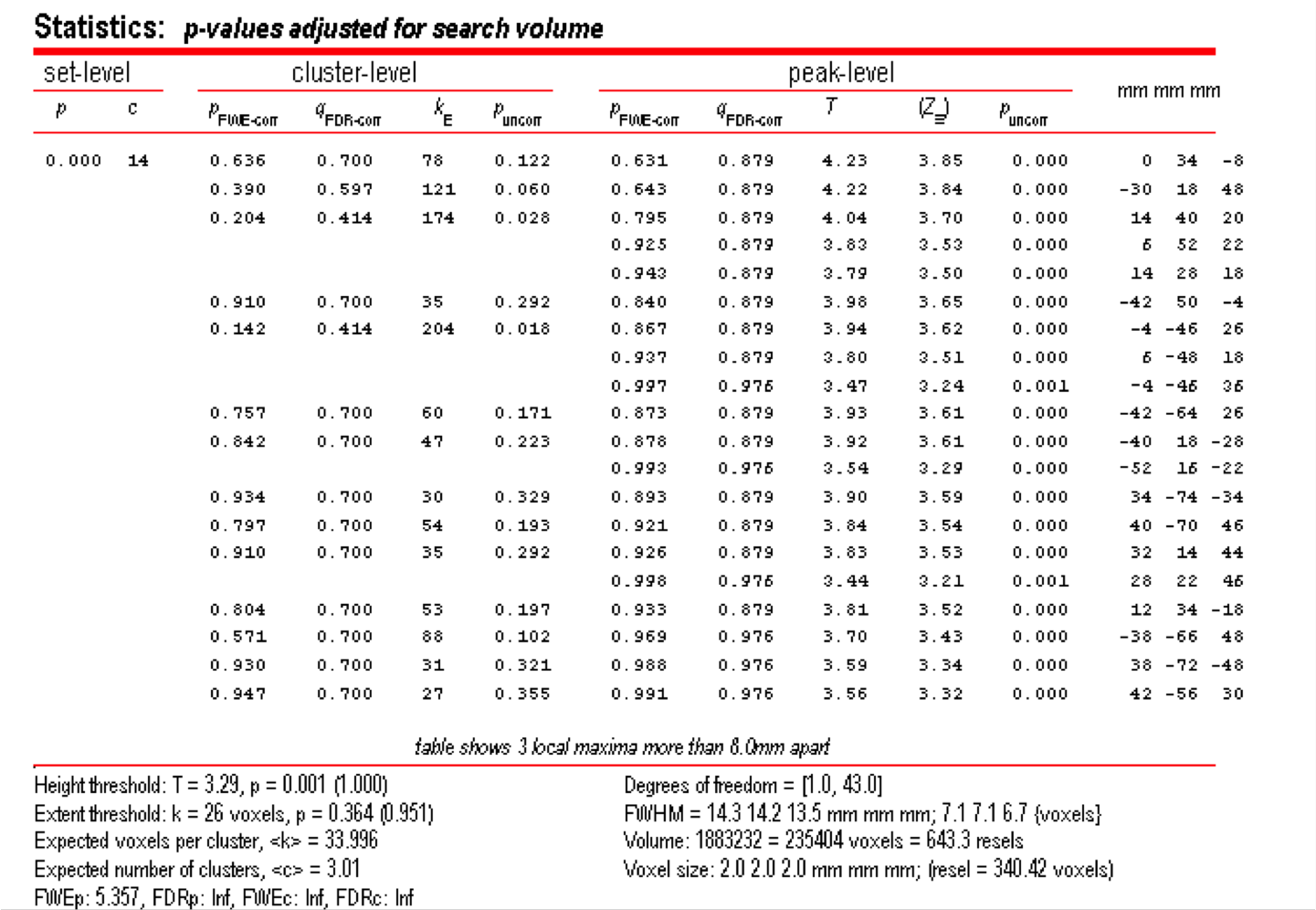


**Figure 3**. Whole-brain analysis results of the ATT group compared to the sham ATT group at T1 when presented with incongruent stimuli

2.2.2 Stroop

When analyzing the “incongruent > congruent” condition, a Stroop interference effect was found during both sessions in the form of increased activation in the left superior and inferior parietal lobe across all subjects, i.e. the ATT and sham ATT group together (see table 2).

Furthermore, we observed increased activation in the left precentral and left postcentral gyri across both groups during T1.

**Table 3**: Significant clusters/peaks during the Stroop task.

| **Location (AAL)** | **Hemisphere** | **x** | **y** | **z** | **Cluster p-value** | **Cluster size** | **p-value** | **T-value (peak voxel)** |
| --- | --- | --- | --- | --- | --- | --- | --- | --- |
| **All subjects, T0 Incongruent > congruent,** *FWE corrected on cluster level* | | | | | | | | |
| Inferior parietal gyrus | L | -38 | -40 | 44 | < 0.001 | 5198 | < 0.00001 | 9.27 |
| Inferior temporal gyrus | L | -48 | -64 | -10 | < 0.001 | 4896 | < 0.00001 | 8.96 |
| Cerebellum | L | -34 | -68 | -54 | < 0.001 | 258 | < 0.00001 | 7.58 |
| Precentral gyrus | L | -44 | 2 | 42 | < 0.001 | 1046 | < 0.00001 | 7.58 |
| Thalamus | L | -14 | -30 | 10 | < 0.001 | 147 | < 0.00001 | 7.20 |
| Middle frontal gyrus | R | 42 | 52 | 18 | < 0.001 | 68 | < 0.00001 | 6.78 |
| Thalamus | L | -6 | -14 | 16 | < 0.001 | 108 | < 0.00001 | 6.77 |
| Thalamus | R | 14 | -10 | 16 | < 0.001 | 136 | < 0.00001 | 6.61 |
| Thalamus | L | -8 | -20 | -10 | 0.001 | 47 | < 0.00001 | 6.60 |
| Supplementary motor area | L | 0 | 16 | 48 | < 0.001 | 485 | < 0.00001 | 6.17 |
| Thalamus | R | 20 | -30 | 14 | 0.011 | 12 | < 0.00001 | 6.04 |
| Inferior frontal gyrus, opercular part | R | 48 | 14 | 28 | 0.003 | 30 | < 0.00001 | 5.93 |
| Temporal pole: superior temporal gyrus | R | 54 | 16 | -4 | 0.001 | 42 | < 0.00001 | 5.85 |
| Angular gyrus | R | 28 | -52 | 36 | 0.007 | 17 | < 0.00001 | 5.77 |
| Calcarine fissure and surrounding cortex | R | 14 | -74 | 18 | 0.002 | 39 | < 0.00001 | 5.72 |
| Inferior parietal gyrus | R | 40 | -48 | 54 | 0.001 | 45 | < 0.00001 | 5.72 |
| Middle frontal gyrus | L | -42 | 48 | 16 | 0.012 | 11 | < 0.00001 | 5.67 |
| Angular gyrus | R | 32 | -62 | 48 | 0.001 | 60 | < 0.00001 | 5.65 |
| Inferior frontal gyrus, triangular part | L | -44 | 42 | 6 | 0.024 | 12 | < 0.00001 | 5.49 |
| Insula | R | 38 | 18 | -8 | 0.016 | 21 | < 0.00001 | 5.48 |
| **All subjects, T1 Incongruent > congruent,** *FWE corrected on Cluster level* | | | | | | | | |
| Inferior parietal gyrus | L | -28 | -48 | 38 | < 0.001 | 2636 | < 0.00001 | 7.80 |
| Precentral gyrus | L | -46 | 8 | 34 | < 0.001 | 922 | < 0.00001 | 6.94 |
| Cerebellum | R | 18 | -60 | -46 | < 0.001 | 213 | < 0.00001 | 6.46 |
| Cerebellum | R | 18 | -52 | -28 | < 0.001 | 196 | < 0.00001 | 6.26 |
| Insula | L | -32 | 18 | 2 | < 0.001 | 90 | < 0.00001 | 5.78 |
| Inferior frontal gyrus, opercular part | R | 50 | 16 | 0 | 0.001 | 71 | < 0.00001 | 5.71 |
| Inferior temporal gyrus | L | -46 | -58 | -14 | 0.003 | 42 | < 0.00001 | 5.63 |
| Cerebellum | R | 10 | -74 | -26 | 0.005 | 30 | < 0.00001 | 5.63 |
| Supplementary motor area | L | -8 | 8 | 54 | 0.014 | 12 | < 0.00001 | 5.43 |
